# Polymicrobial sepsis impairs bystander recruitment of effector cells to infected skin despite optimal sensing and alarming function of skin resident memory CD8 T cells

**DOI:** 10.1371/journal.ppat.1006569

**Published:** 2017-09-14

**Authors:** Derek B. Danahy, Scott M. Anthony, Isaac J. Jensen, Stacey M. Hartwig, Qiang Shan, Hai-Hui Xue, John T. Harty, Thomas S. Griffith, Vladimir P. Badovinac

**Affiliations:** 1 Interdisciplinary Program in Immunology, University of Iowa, Iowa City, Iowa, United States of America; 2 Department of Pathology, University of Iowa, Iowa City, Iowa, United States of America; 3 Department of Microbiology, University of Iowa, Iowa City, Iowa, United States of America; 4 Microbiology, Immunology and Cancer Biology Ph.D. Program, University of Minnesota, Minneapolis, Minnesota, United States of America; 5 Center for Immunology, University of Minnesota, Minneapolis, Minnesota, United States of America; 6 Department of Urology, University of Minnesota, Minneapolis, Minnesota, United States of America; 7 Minneapolis VA Health Care System, Minneapolis, Minnesota, United States of America; Cincinnati Children's Hospital Medical Center, UNITED STATES

## Abstract

Sepsis is a systemic infection that enhances host vulnerability to secondary infections normally controlled by T cells. Using CLP sepsis model, we observed that sepsis induces apoptosis of circulating memory CD8 T-cells (T_CIRCM_) and diminishes their effector functions, leading to impaired CD8 T-cell mediated protection to systemic pathogen re-infection. In the context of localized re-infections, tissue resident memory CD8 T-cells (T_RM_) provide robust protection in a variety of infectious models. T_RM_ rapidly ‘sense’ infection in non-lymphoid tissues and ‘alarm’ the host by enhancing immune cell recruitment to the site of the infection to accelerate pathogen clearance. Here, we show that compared to pathogen-specific T_CIRCM_, sepsis does not invoke significant numerical decline of Vaccinia virus induced skin-T_RM_ keeping their effector functions (e.g., Ag-dependent IFN-γ production) intact. IFN-γ-mediated recruitment of immune cells to the site of localized infection was, however, reduced in CLP hosts despite T_RM_ maintaining their ‘sensing and alarming’ functions. The capacity of memory CD8 T-cells in the septic environment to respond to inflammatory cues and arrive to the site of secondary infection/antigen exposure remained normal suggesting T-cell-extrinsic factors contributed to the observed lesion. Mechanistically, we showed that IFN-γ produced rapidly during sepsis-induced cytokine storm leads to reduced IFN-γR1 expression on vascular endothelium. As a consequence, decreased expression of adhesion molecules and/or chemokines (VCAM1 and CXCL9) on skin endothelial cells in response to T_RM_-derived IFN-γ was observed, leading to sub-optimal bystander-recruitment of effector cells and increased susceptibility to pathogen re-encounter. Importantly, as visualized by intravital 2-photon microscopy, exogenous administration of CXCL9/10 was sufficient to correct sepsis-induced impairments in recruitment of effector cells at the localized site of T_RM_ antigen recognition. Thus, sepsis has the capacity to alter skin T_RM_ anamnestic responses without directly impacting T_RM_ number and/or function, an observation that helps to further define the immunoparalysis phase in sepsis survivors.

## Author summary

Infectious pathogens are generally relegated within barrier tissues; however, when infections enter the bloodstream the host enters a septic state that (when severe enough) can lead to widespread tissue damage and death. After resolution of acute stage of sepsis, patients often display enhanced susceptibility to secondary infections resulting from quantitative and qualitative alterations in the immune response. Previously, we and others have shown that sepsis dramatically reduces the number and function of memory CD8 T cells within the host, contributing to the state of immunoparalysis early after sepsis induction. The present study directly examines the impact of sepsis on tissue resident memory CD8 T cells (T_RM_) that are restricted to barrier tissues and provide protection to localized infections. In contrast to circulating memory T cells found within lymphoid tissues, the number and function of T_RM_ within non-lymphoid peripheral tissue (such as the skin) was maintained during sepsis suggesting a protective niche for memory CD8 T cells within barrier tissues of septic hosts. However, the capacity of T_RM_ to provide protection upon re-infection was severely diminished in septic hosts, which was attributed to a sepsis-induced lesion in T_RM_-extrinsic factors. Thus, this report supports the notion that sepsis has the capacity to influence host response to pathogen re-infection either by directly influencing memory CD8 T cell populations or by preventing other cell types to properly recognize localized pathogen-induced alarming signals delivered by resident memory CD8 T cells.

## Introduction

Sepsis is the result of a systemic infection that elicits a cytokine storm characterized by host tissue damage following production of pro- and anti-inflammatory cytokines. Globally, there are 31.5 million cases and 5.3 million deaths from sepsis annually. Interestingly, most mortality occurs after resolution of the relatively brief acute phase of sepsis and is associated with enhanced susceptibility to secondary infections usually eradicated by a normal functioning immune system [1,2,3,4,5]. Observations of T cell apoptosis in septic patients originally provided insight to a potential underlying lesion in T cell-mediated immunity during the state of immunoparalysis after sepsis induction [6,7].

Using the cecal ligation and puncture (CLP) experimental model of sepsis induction we previously determined that sepsis reduces the number of naïve CD4 and CD8 T cells and impairs primary T cell responses to a variety of acute (lymphocytic choriomeningitis virus (LCMV) Armstrong, *Listeria monocytogenes*, *Candida albicans*) and chronic (LCMV clone-13) infections [8,9,10,11]. Polymicrobial sepsis also affects infection and/or vaccine-induced memory CD8 T cell responses by inducing apoptosis of pre-formed memory CD8 T cells and impairing the Ag-dependent and—independent memory CD8 T cell functions (e.g., functional avidity and IFN-γ production upon heterologous infection, respectively) [10,12,13]. In addition to these T cell-intrinsic factors affected in the post-septic host, deficiencies in T cell-extrinsic factors [e.g., dendritic cells (DC) capacity to provide ‘signal 3’ cytokines to promote optimal T cell responses], severely restrict the generation of productive T cell responses [14,15]. These reports suggest contributing mechanisms that define the immunoparalysis phase of sepsis leading to increased susceptibility to secondary infections normally controlled by T cells.

Most (if not all) of the prior reports describing the impact of sepsis on naïve or memory T cells have focused on cells in peripheral blood and/or secondary lymphoid organs (SLO). However, vaccination or infection can generate two distinct populations of memory CD8 T cells distinguished by their unique migration capacity and transcription profiles [16]. Circulating memory CD8 T cells (T_CIRCM_) circulate throughout the body in the peripheral blood and SLO searching for cognate Ag, while tissue resident memory CD8 T cells (T_RM_) remain localized in previously-infected host barrier tissues (e.g. skin, lung, gut) commonly exploited by pathogens to mediate host entry [17]. T_RM_ positioned in non-lymphoid tissue (NLT) survey their environment by interacting with antigen-presenting cells (APC) and rapidly facilitate Ag-dependent ‘sensing’ of infection and ‘alarming’ the host, creating a tissue-wide pathogen alert to rapidly orchestrate anamnestic responses [18,19,20,21]. Mechanistically, upon Ag recognition, T_RM_-derived IFN-γ enhances expression of chemokines and adhesion molecules on vascular endothelium to promote bystander recruitment of circulating Ag-experienced T and B cells to the infected tissue to participate in rapid pathogen clearance. Thus, a critical interplay exists between T_RM_ and T_CIRCM_ in providing optimal protection upon pathogen re-encounter [18,19]. The contribution of T_RM_ in mediating protection against pathogen re-challenge has been demonstrated in a variety of tissues including the skin [20,22,23,24], female reproductive tract [18,25], and lung [26,27]. Despite the observed and now appreciated importance of T_RM_ in controlling localized infections, the impact of sepsis on the quantity (number) and quality (capacity to perform Ag-dependent ‘sensing’ and ‘alarming’ function) of CD8 T_RM_ has not been rigorously defined.

To address this knowledge gap, CD8 T_CIRCM_ and skin, lung, and/or small intestine T_RM_ were generated in mice through vaccinia virus (VacV; skin), LCMV (lung and small intestine), or influenza (lung) infections and analyzed after CLP or sham surgery. Our data show sepsis reduced the number and Ag-dependent function (production of IFN-γ) of T_CIRCM_ to a greater extent than T_RM_ of the same Ag-specificity. Despite optimal ‘sensing’ and ‘alarming’ function of skin T_RM_, septic hosts displayed tremendous impairment in bystander T and B cell recruitment to the skin upon T_RM_ activation, which was not solely attributed to numerical loss of these populations in the circulation after sepsis induction. Instead, the vascular endothelium of septic hosts had reduced IFN-γ receptor expression that correlated with reduced expression of IFN-γ inducible proteins on vascular endothelium after skin T_RM_ activation. Lastly, the observed impairments in the T_RM_-mediated anamnestic response greatly reduced the protective capacity of skin T_RM_ to homologous infection in immune mice. In total, our data show the sepsis-induced impairments in skin T_RM_-mediated protection against secondary infection was driven by T_RM_-extrinsic factors. Therefore, sepsis modulates host response to pathogen re-infection either by directly influencing memory CD8 T cell populations or by preventing other cell types to properly respond to pathogen-induced alarming signals delivered by resident memory CD8 T cells in barrier tissues.

## Results

### Sepsis reduces number of T_CIRCM_ to a greater degree than T_RM_ of the same Ag-specificity

The level of CD8 T cell-mediated protection to infection depends on the number of memory CD8 T cells present during pathogen re-challenge [28,29]. Recently, we showed that sepsis reduces the number of pre-existing CD8 T_CIRCM_ and impairs CD8 T cell-mediated protection to systemic bacterial and viral infections [12]. CD8 T_CIRCM_ are only part of the memory CD8 T cell compartment, as CD8 T_RM_ are now recognized to be critical in providing protection against localized infections in a variety of barrier tissues (e.g. skin and lung) [22,24,26].

To generate circulatory and tissue-resident memory CD8 T cells, a low number of naïve Thy1.1 P14 cells specific for LCMV-derived GP_33_ epitope were transferred into Thy1.2 C57Bl/6 (B6) mice followed by VacV-GP_33_ infection of the left ear [30]. At a memory time point (d 30+ post infection), CD45.2 mAb was injected i.v. to distinguish T_RM_ (CD45.2-) from T_CIRCM_ (CD45.2+; S1A Fig) [31,32]. VacV-GP_33_ infection generated a memory P14 population in the left ear protected from CD45.2 mAb recognition and expressing CD103, a marker of skin T_RM_ [33]. Consistent with previous data, CD8 T_RM_ also accumulate in distal locations from the primary site of skin infection, such as the contralateral right ear, but at greatly reduced number (S1B Fig) [22,24,34]. In a similar experimental approach using systemic LCMV infection, skin T_RM_ were not detected in the left ear (S1C and S1D Fig), further supporting the premise that skin T_RM_ formation is dependent on the route of infection and specific pathogen [35].

**Fig 1:**
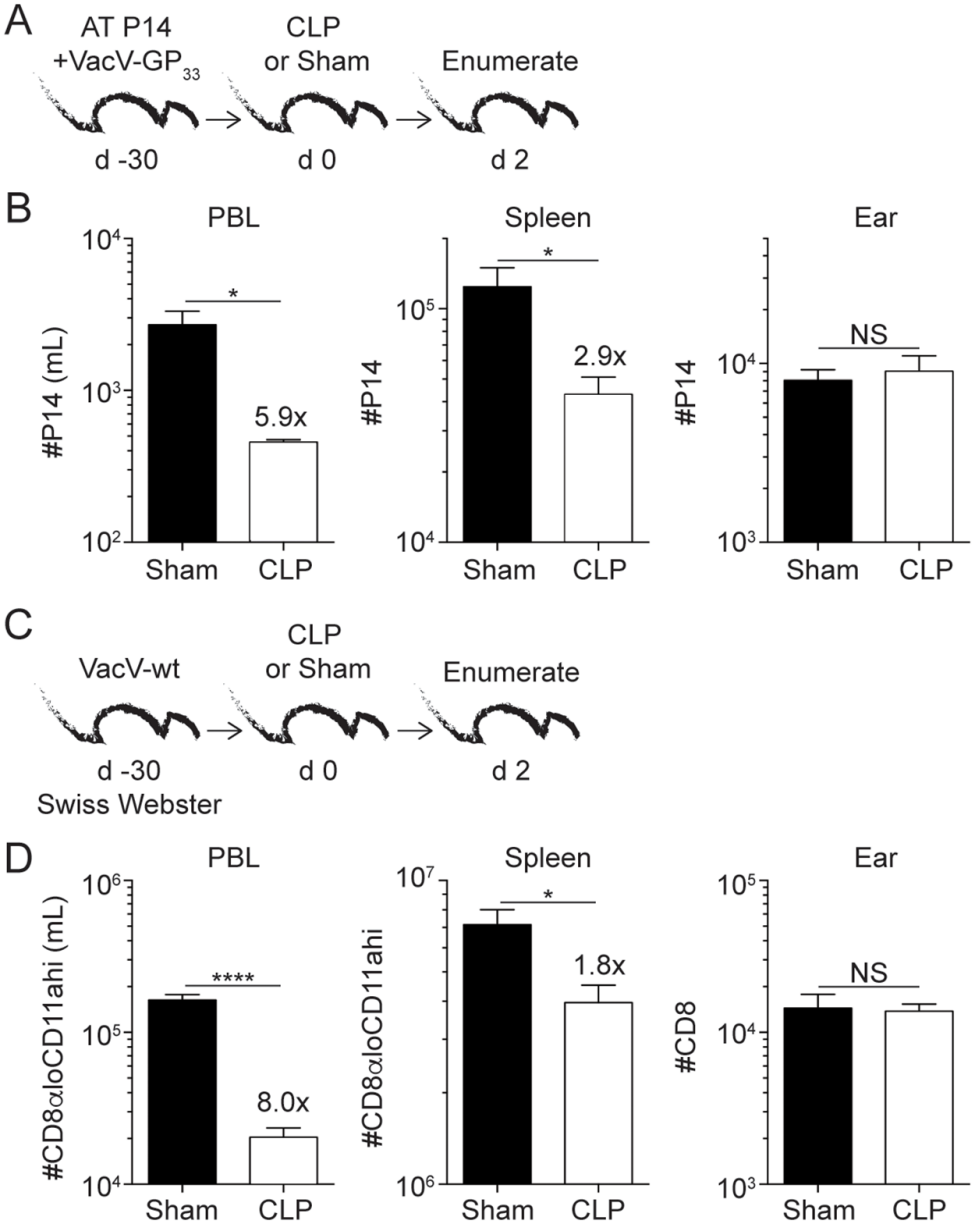
Sepsis reduces the number of CD8 T_CIRCM_ to a greater extent than skin T_RM_ of the same Ag-specificity. (A) Experimental Design. 5 x 10^3^ naïve P14 CD8 T cells (Thy1.1) were adoptively transferred into C57Bl/6 recipients (Thy1.2) followed by VacV-GP_33_ infection of the left ear. After 30 days mice underwent CLP or sham surgery and two days later mice received intravascular injection of CD45.2 mAb. Tissues of interest were harvested three minutes later. (B) Enumeration of P14 T_CIRCM_ in peripheral blood and spleen and P14 skin T_RM_ (CD45.2-CD103+) in the left ear two days after surgery. (C) Experimental Design. Swiss Webster outbred mice received wild type VacV infection of the left ear. After 30 days mice underwent CLP or sham surgery, and two days later tissues of interest were harvested and cells enumerated. (D) Enumeration of Ag-experienced (CD8α^lo^CD11a^hi^) T_CIRCM_ in peripheral blood and spleen and CD8 skin T_RM_ (CD45.2-CD103+) in the left ear two days after surgery. Data are representative of two independent experiments with (A-B) 4 mice per group or (C-D) 7–9 mice per group. NS = not significant; * = p<0.05; **** = p<0.0001. Error bars represent the standard error of the mean.

With a system in place to generate T_RM_, we next examined the impact of sepsis on pre-existing Ag-specific skin T_RM_. VacV-GP_33_-immune mice underwent cecal ligation and puncture (CLP) or sham surgery and 2 days later memory P14 cells enumerated (Fig 1A). As expected, sepsis reduced the number of P14 T_CIRCM_ in the blood and spleen by 5.9- and 2.9-fold, respectively. In contrast, the number of P14 T_RM_ (gated and defined as cells not labeled with intravascular injected antibody (CD45.2 negative) and CD103+) in the skin did not change after sepsis induction (Fig 1B). Of note, most of the T_CIRCM_ cell death in the peripheral blood occurs during the first 48 hours after sepsis induction coinciding with duration of sepsis-induced cytokine storm (S2A–S2C Fig). However, to formally prove that sepsis does not influence T_RM_ with delayed kinetics compared to T_CIRCM_ both memory CD8 T cell populations were analyzed 4 days post sepsis induction (S2D–S2F Fig). Results clearly showed that ear T_RM_ are still numerically intact compared to sham counterparts suggesting apoptosis of the T_RM_ population is not delayed in this system (S2D–S2F Fig). Together, these data suggest T_CIRCM_ are more susceptible to the sepsis-mediated cues responsible for deletion than skin T_RM_ of the same Ag-specificity.

Inbred mouse strains facilitate tracking an Ag-specific CD8 T cell population with known stimulation history; however, inbred mice are genetically homogenous and do not necessarily capture the genetic diversity associated with a human population. To determine the impact of sepsis on the number of skin T_RM_ in a genetically heterogeneous population, we performed sham or CLP surgery on VacV-immune outbred Swiss Webster mice (Fig 1C). Similar to what was observed with the inbred B6 mice, sepsis significantly reduced the number of CD8 T_CIRCM_ (distinguished using surrogate activation marker of memory CD8 T cells, CD8α^lo^CD11a^hi^) [36,37] in the peripheral blood and spleen but not skin CD8 T_RM_. (Fig 1D). Collectively, the data in Fig 1 show that sepsis leads to significant numerical loss of CD8 T_CIRCM_ in peripheral blood and SLO but little-to-no numerical alterations of skin T_RM_ counterparts regardless of the genetics of the hosts examined.

Using different pathogens and routes of infection we generated T_RM_ in other NLT (e.g. lung and small intestine) and determined the impact of sepsis on the number of T_RM_. To test the impact of sepsis on lung T_RM_, B6 mice received an adoptive transfer of naïve P14 cells followed by intranasal IAV PR8-GP_33_ infection. The mice underwent CLP or sham surgery 35 days later, and CD45.2 mAb was injected i.v. and memory P14 cells enumerated after 2 days (S3A Fig). Sepsis increased the representation of CD45.2- memory P14 cells in the lung parenchyma and airways (S3B and S3C Fig), which was primarily attributed to numerical loss of CD45.2+ lung P14 T_CIRCM_ (S3D Fig) with negligible numerical impact on lung T_RM_ (S3E Fig). In the same influenza-immune hosts, splenic P14 T_CIRCM_ underwent significant numerical loss after sepsis induction (S3F Fig). These data suggest sepsis reduces the number of influenza-specific T_CIRCM_ to a greater extent than influenza-specific T_RM_ in the lung. Similarly, in a repeated experiment we determined the impact of sepsis on the number of total CD8 T cells in influenza-immune mice. (S3G Fig). CLP hosts had an increased representation of CD45.2- lung CD8 T cells compared to sham controls (S3H–S3J Fig), largely resulting from the loss of CD45.2+ CD8 T cells in the lungs (S3K Fig). To rule out the possibility that the numerical loss of CD45.2+ CD8 T cells in the CLP-treated mice was a result of cell redistribution within the host, we found a significant increase in the frequency of CD45.2+ lung vasculature CD8 T cells with activated caspase-3/7 in CLP- versus sham-treated mice (S3L–S3N Fig) suggesting sepsis leads to apoptosis of circulatory memory CD8 T cells.

Lastly, we examined the impact of sepsis on the number of lung and small intestine T_RM_ in LCMV-Armstrong infected mice. Naïve P14 cells were transferred into B6 mice before i.p. infection with LCMV-Armstrong. CLP or sham surgery was performed 30 days later, and tissues were harvested after 2 days (S4A Fig). Similar to previous models, sepsis increased the representation of lung and small intestine CD45.2- P14 cells compared to their CD45.2+ counterparts (S4B–S4D Fig) which attributed with a significant decline in the number of lung P14 T_CIRCM_ (S4E Fig).

Taken together, using three infection models (VacV, influenza, and LCMV) to generate CD8 T_RM_ populations in multiple barrier tissues (skin, lung, and small intestine) before CLP or sham surgery, our data demonstrate sepsis greatly reduces the total number of pre-existing memory CD8 T cells. However, the observed numerical loss after sepsis is primarily occurring in the CD8 T_CIRCM_ population leaving the tissue-embedded CD8 T_RM_ population numerically intact.

### CD8 skin T_RM_ maintain Ag-dependent ‘sensing’ and ‘alarming’ function after sepsis induction

**Fig 2:**
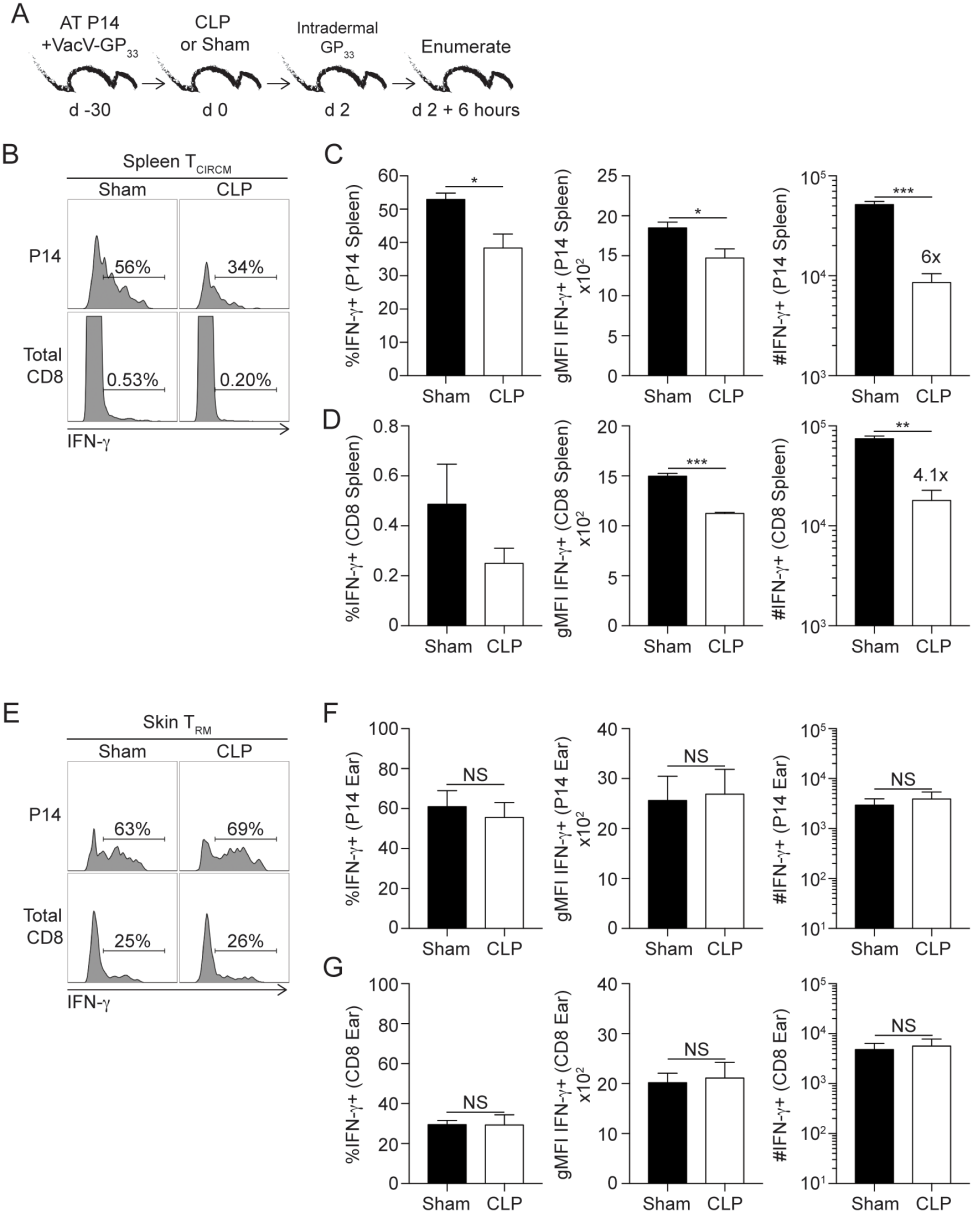
Skin CD8 T_RM_ maintain Ag-dependent ‘sensing and alarming’ function after sepsis induction. (A) Experimental Design. 10^4^ naïve P14 CD8 T cells (Thy1.1) were adoptively transferred into C57Bl/6 recipients (Thy1.2) followed by VacV-GP_33_ infection of the left ear. After 30 days mice underwent CLP or sham surgery and two days later mice received 50μg of GP_33-41_ peptide via intradermal injection in the left ear. After 6 hours of *in vivo* peptide stimulation, mice received intravascular injection of CD45.2 mAb. Tissues of interest were harvested three minutes later. (B) Representative gating and frequency of IFN-γ producing P14 T_CIRCM_ and total CD8 T cells in sham and CLP hosts. (C) Summary data of frequency and number of P14 T_CIRCM_ IFN-γ production. (D) Summary data of frequency and number of total CD8 T cells IFN-γ production. (E) Representative gating and frequency of IFN-γ producing P14 and CD8 skin T_RM_ in the left ear from sham and CLP hosts. (F) Summary data of frequency and number of P14 T_RM_ IFN-γ production. (G) Summary data of frequency and number of total CD8 T_RM_ IFN-γ production. Data are representative of five independent experiments with at least 3 mice per group per experiment. NS = not significant; * = p<0.05; ** = p<0.01; *** = p<0.001. Error bars represent the standard error of the mean.

We previously reported that, in addition to the numerical loss of cells, sepsis also reduces the Ag-dependent function (e.g., production of effector cytokines or secondary proliferative expansion in numbers following Ag re-encounter) of surviving CD8 T_CIRCM_ further contributing to the observed impairment in CD8 T cell-mediated immunity [12]. Upon homologous infection and recognition of cognate Ag, T_RM_-derived effector cytokines (e.g., IFN-γ) invoke a tissue-wide state of pathogen alert and facilitate bystander recruitment of memory B and T cells to participate in pathogen clearance [18,19,20]. Thus, we next determined the impact of sepsis on Ag-dependent ‘sensing’ and ‘alarming’ function of skin T_RM_ *in vivo*. VacV-immunized mice received CLP or sham surgery 30 days after primary infection. Two days later, mice received *in vivo* GP_33_ peptide stimulation and 6 hours later tissues were harvested and intracellular cytokine stain (ICS) for IFNγ performed directly *ex vivo* without further stimulation and/or inhibition of protein transport with Brefeldin A (Fig 2A) [34]. Importantly, *in vivo* intradermal peptide injection activated both T_RM_ and T_CIRCM_, allowing us to determine the effect of sepsis on Ag-dependent functions of both CD8 T_RM_ and T_CIRCM_ in the same host. Sepsis reduced the frequency, per-cell production, and number of IFN-γ producing T_CIRCM_ in the spleen (Fig 2B–2D). In contrast, skin T_RM_ in septic hosts retained their ability to sense cognate Ag and respond with IFN-γ production to the same degree as in sham-treated mice (Fig 2E–2G). Although it is unlikely that IFN-γ production detected by memory CD8 T cells occurs *in vitro* during the relatively short period needed to process and stain ear/spleen cells, it is formally possible that residual antigen (if any) can prime or facilitate IFN-γ production by memory CD8 T cells present in the cell suspension. To directly test this, CFSE-labeled ‘sensor’ cells (memory GP_33_-specific P14 CD8 T cells obtained from LCMV immune mice) were added to the tubes in which organs were collected (S5A Fig). Thus, ‘sensor’ cells would be with tissue cells all the time during sample processing and staining. Importantly, ‘sensor’ cells did not produce any measurable IFN-γ suggesting IFN-γ production by T_CIRCM_ and T_RM_ cells occurred as a consequence of Ag recognition *in situ* (S5B–S5D Fig). Finally, sepsis did not change the phenotype (subset composition) and cytolytic potential of ear CD8 T_RM_ compared to sham controls (S6 Fig). Therefore, T_RM_ cells at periphery showed unexpected resistance to sepsis.

### Sepsis impairs bystander recruitment of circulating B and T cells to the skin upon activation of skin CD8 T_RM_

**Fig 3:**
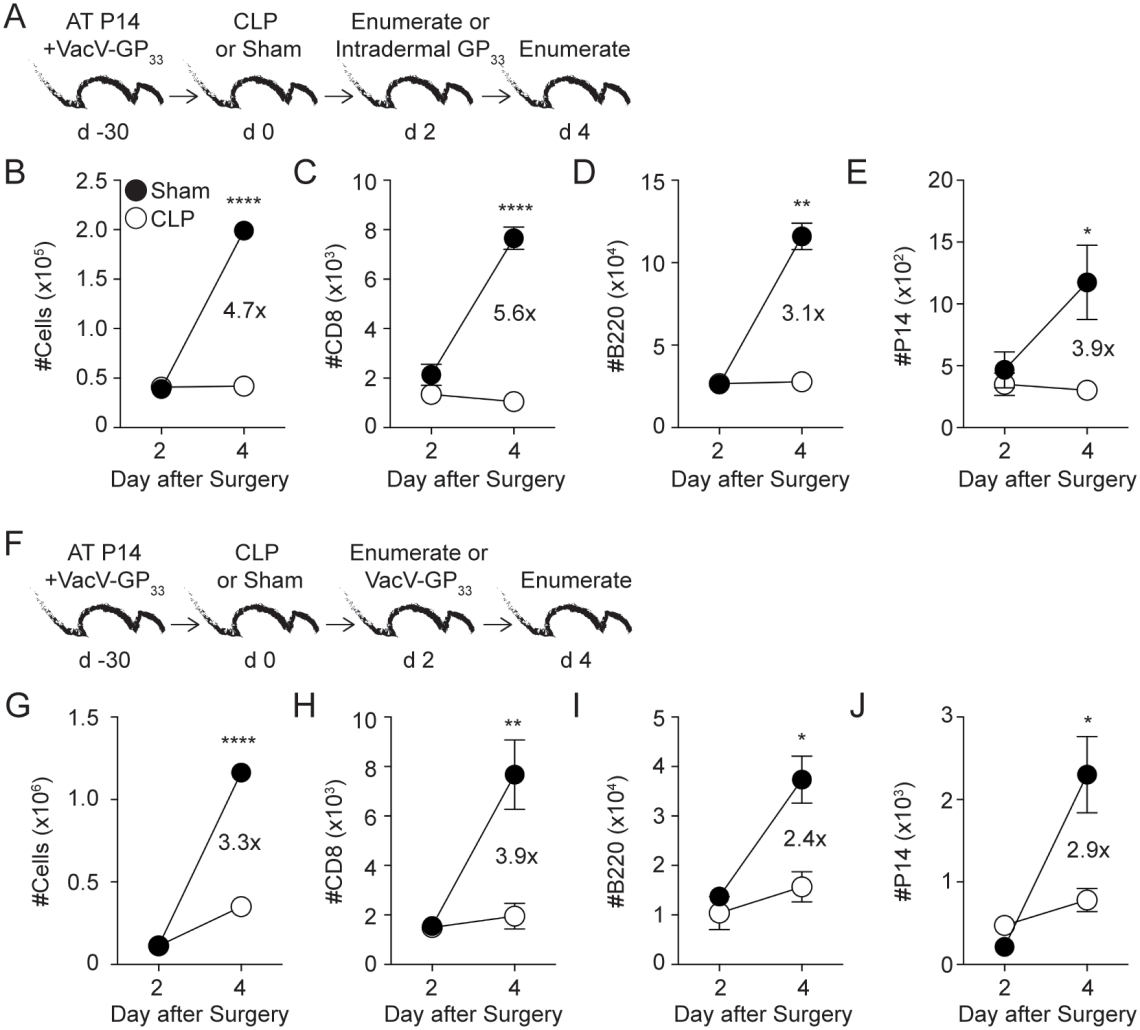
Sepsis impairs bystander recruitment of circulating cells to the skin upon Ag-dependent activation of skin CD8 T_RM_. (A, F) Experimental Designs. 10^4^ naïve P14 CD8 T cells (Thy1.1) were adoptively transferred into C57Bl/6 recipient hosts (Thy1.2) followed by VacV-GP_33_ infection of the left ear. After 30 days mice underwent CLP or sham surgery. Mice received an intravascular injection of CD45.2 mAb 2 days later, and cells within the left ear were enumerated or mice received intradermal injection of 50μg of GP_33-41_ peptide (A) or homologous VacV-GP_33_ infection (F) in the left ear. The peptide injected or VacV infected mice then received an intravascular injection of CD45.2 mAb 2 days later and cells within the left ear were enumerated. (B) Total cell count and number of (C) CD8+CD45.2-, (D) B220+CD8-CD45.2- or (E) P14 CD45.2- cells before and after skin T_RM_-mediated bystander recruitment to the ear, day 2 and 4, respectively. (G) Total cell count and number of (H) CD8+ CD45.2-, (I) B220+CD8- CD45.2- or (J) P14 CD45.2- cells before and after VacV re-challenge (day 2 and 4, respectively). Data are representative of two independent experiments with 3–5 mice per group per experiment. * = p<0.05; ** = p<0.01; **** = p<0.0001. Error bars represent the standard error of the mean.

T_RM_-derived IFN-γ induces the expression of chemokines and adhesion molecules on local vascular endothelium to promote bystander recruitment of Ag-experienced T and B cells to the site of T_RM_ activation [18,19]. Since sepsis had no impact on the ‘sensing’ and ‘alarming’ Ag-dependent function of skin T_RM_, we did not expect to see any modulation in bystander immune cell recruitment in septic hosts upon Ag/pathogen re-encounter at the local site. To test this, VacV-immune mice underwent CLP or sham surgery 30 days after primary VacV infection. The mice received CD45.2 mAb i.v. injection 2 days later to determine baseline cellularity, and cells within the ear were enumerated. Additional cohorts of mice received *in vivo* GP_33_ peptide stimulation in the ear, followed by enumerating the recruited cells from the circulation 2 days later (Fig 3A) [18]. Surprisingly, sepsis impaired the recruitment of circulating cells to the site of skin T_RM_ activation resulting in a 4.7-fold reduction in ear cellularity (compared to sham-treated mice), which was attributed to 5.6-, 3.1-, and 3.9-fold decreases in number of CD8+ and B cells (CD8-/B220+) and P14 cells in the ear, respectively (Fig 3B–3E). Next, we tested the capacity of skin T_RM_ to recruit circulating memory B and T cells to the skin upon homologous VacV infection (Fig 3F). Upon VacV re-challenge, CLP hosts had 3.9-, 2.4-, and 2.9-fold reductions in the number of CD8+, B cells (CD8-/B220+) and P14 cells, respectively, at the site of re-infection compared to sham counterparts (Fig 3G–3J). Thus, despite proper number and optimal Ag-dependent ‘sensing’ and ‘alarming’ functions of skin T_RM_, sepsis impairs bystander recruitment of circulating T and B cells to the site of skin T_RM_ activation.

### Capacity of CD8 T_CIRCM_ cells to follow inflammatory cues and migrate to the inflamed tissue is not influenced by sepsis

**Fig 4:**
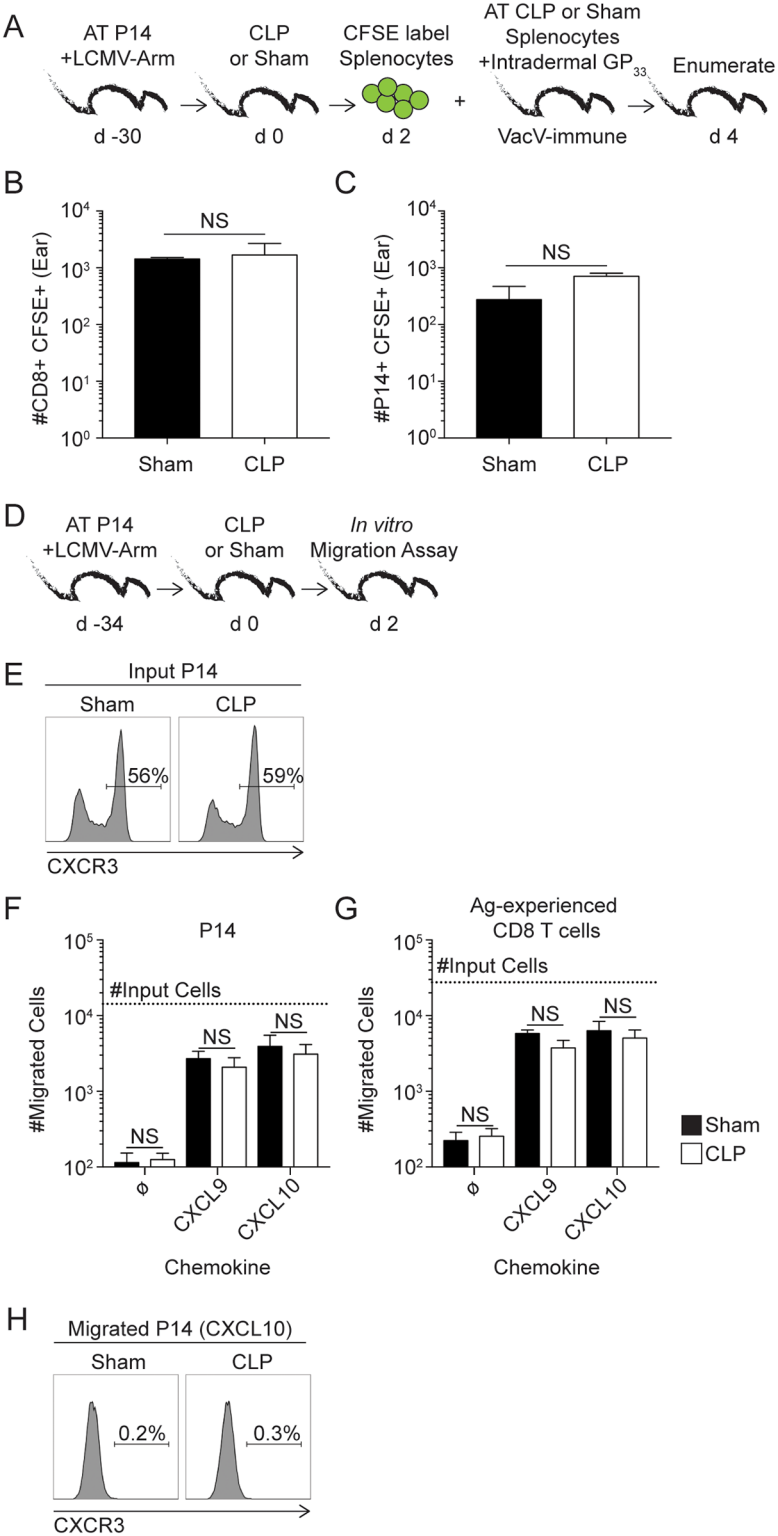
Capacity of circulatory memory CD8 T cells to follow inflammatory cues and migrate to the inflamed tissue is not influenced by sepsis. (A) Experimental design. Two days after CLP or sham surgery splenocytes from LCMV-Arm immune P14 chimera mice were harvested, CFSE labeled, and transferred into VacV-GP_33_ immune recipients that contained P14 T_RM_ before *in vivo* peptide stimulation of the skin. (B) Number of CD8+ CD45.2- CFSE+ and (C) P14 CD45.2- CFSE+ cells in the ear 48 hours after skin T_RM_ activation. (D) Experimental design. Splenocytes for *in vitro* migration assay were harvested from LCMV immune P14 chimera mice 2 days post CLP or sham surgery. (E) Representative histograms of CXCR3 expression on P14 T_CIRCM_ from sham or CLP hosts used in transwell migration assay. Number of (F) P14 and (G) Ag-experienced CD8 T cells (CD8α^lo^CD11a^hi^) that migrated into transwell receiving plate in response to medium alone or in response to CXCL9, or CXCL10 chemoattractant stimulation. Dashed line represents the total number of P14 or Ag-experienced CD8 T cells that were initially placed in the top chamber of the transwell system. (H) Representative histogram of CXCR3 expression on P14 T_CIRCM_ from sham or CLP hosts that migrated to receiving plate in response to CXCL10. Data representative of two similar experiments with at least 3 mice per group. NS = not significant. Error bars represent the standard error of the mean.

One obvious explanation for the observed impairment in bystander recruitment was the sepsis-induced numerical loss of circulating memory B and T cells, effectively limiting the number of cells that could potentially respond to inflammatory cues and travel to the site of infection. However, it is also formally possible that sepsis or the post-septic environment changes the ability of circulatory effector cells (in this case memory CD8 T cells) to respond to inflammatory cues and arrive at the place of re-infection. To directly test this, equal number of splenocytes containing memory CD8 T cells obtained from LCMV-immune P14 chimera mice 2 days after sepsis induction were CFSE labeled and transferred into congenic Vacv-GP_33_-immune P14 chimera mice (Fig 4A). Ears of all recipient mice were treated with GP_33_ peptide to trigger sensing and alarming function of T_RM_ and the number of CFSE-labeled CD8+ or P14 cells enumerated in the ears 2 days later. Interestingly, spending 2 days in the CLP environment does not change the ability of memory CD8 T cells to migrate into the normal skin (Fig 4A–4C) suggesting that sepsis does not change capacity of remaining memory CD8 T cells to follow inflammatory cues *in vivo*.

To further boost this observation and provide mechanistic insight into the notion that T cells exposed to septic environment retain ability to respond to inflammation, T_CIRCM_ were obtained from sham or CLP LCMV-immune hosts 2 days after surgery and their migratory capacity to CXCR3 ligands (CXCL9 and 10) [38, 39] was analyzed directly *ex vivo* in a transwell system (Fig 4D). As suggested in S2 Fig where the phenotype of cells obtained from sham or CLP hosts was compared, sepsis also did not change the expression of CXCR3 on P14 T_CIRCM_ cells used in this assay (Fig 4E). No discernable differences were observed in the migratory capacity of memory P14 or bulk Ag-experienced CD8 T cells obtained from sham or CLP hosts in response to CXCL9 or CXCL10 stimuli (Fig 4F and 4G). Consistent with published data [40,41], all the migrated P14 cells lost the expression of CXCR3 strongly suggesting CXCL9/10-dependent migration (Fig 4H). Taken together, the data shown here suggest sepsis does not influence memory CD8 T cell capacity to recognize inflammatory cues and relocate to the site of inflammation.

### Impairments in bystander recruitment upon skin CD8 T_RM_ activation are not solely due to sepsis-induced numerical loss of circulating cells

**Fig 5:**
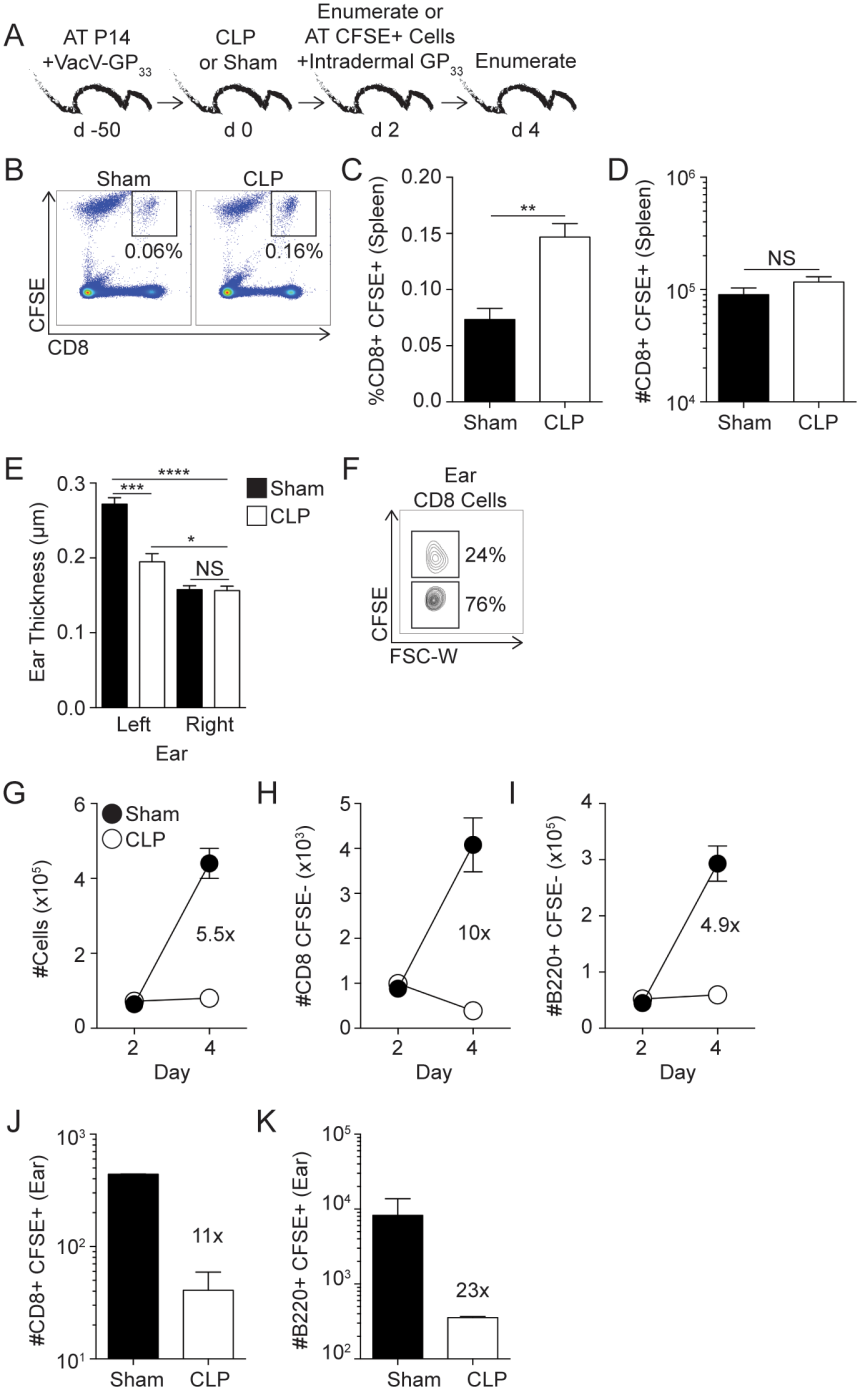
Impairments in bystander recruitment upon skin CD8 T_RM_ activation is not solely due to sepsis-induced numerical loss of circulating cells. (A) Experimental Design. 8 x 10^3^ Naïve P14 CD8 T cells (Thy1.1) were adoptively transferred into C57Bl/6 recipient hosts (Thy1.2) followed by VacV-GP_33_ infection of the left ear. After 50 days mice underwent CLP or sham surgery. Mice received intravascular injection of CD45.2 mAb 2 days later and three minutes later cells within the left ear were enumerated to determine baseline cellularity. Another group of VacV-immune mice received adoptive transfer of 14 × 10^6^ CFSE-labeled splenocytes from LCMV-immune donor mice and intradermal injection of 50μg of GP_33-41_ peptide in the left ear performed to activate GP_33_-specific skin T_RM_. Mice received intravascular injection of CD45.2 mAb 2 days later. Tissues of interest were harvested 3 minutes later and analyzed. (B) Representative gating of CFSE+ CD8 T cells in the spleen of sham and CLP hosts. Frequency (C) and number (D) of adoptively transferred CFSE+ CD8 T cells within the spleen of CLP and sham mice 4 days after surgery. (E) Ear thickness of left and right ears two days after *in vivo* peptide stimulation of skin T_RM_. (F) Representative gating of CFSE+ and CFSE- CD8 T cells within the left ear. (G) Total cells in left ear and number of (H) CD8+CD45.2-CFSE- and (I) B220+CD8-CD45.2-CFSE- cells of the left ear on day 2 and 4 after surgery. Number of CD45.2-CFSE+ (J) CD8 and (K) B220+ cells within the left ear day 4 after surgery. Data are representative of two independent experiments with 2–4 mice per group per experiment. Two-way ANOVA statistical test was performed for panel E. NS = not significant; * = p<0.05; ** = p<0.01; *** = p<0.001; **** = p<0.0001. Error bars represent the standard error of the mean.

To determine the extent to which the lesion in bystander recruitment was due to sepsis-induced numerical loss of circulating cells or if T cell-extrinsic factor(s) contributed to the defect observed, VacV-immune mice underwent CLP or sham surgery and cells within left ear were enumerated 2 days later. A companion cohort of mice received equal numbers of CFSE-labeled splenocytes from LCMV-immune donor mice, followed by *in vivo* GP_33_ peptide injection in the left ear to activate skin T_RM_ and induce the recruitment of bystander host (CFSE-) or transferred (CFSE+) cells (Fig 5A). Due to the lymphopenic environment of septic hosts, the CD8+CFSE+ donor cells comprised a significantly higher frequency of the cells within the spleen compared to sham controls (Fig 5B and 5C) but a similar number of these cells were present in the spleens of both groups of mice (Fig 5D).

Activation of skin T_RM_ will lead to an increase in the thickness of the ear pinna, indicating a localized anamnestic response [34]. We noted a significant reduction in ear thickness in the CLP-treated mice following skin T_RM_ activation compared to sham controls, providing further evidence that sepsis impairs the recruitment of bystander cells to the site of skin T_RM_ activation (Fig 5E). In addition to assessing the general response at the level of ear thickness, this experimental design also facilitated the specific quantitation of host (CFSE-)- and donor (CFSE+)-derived bystander cell recruitment of cells in the same animal (Fig 5F). Similar to Fig 3, CLP-treated mice showed a significant reduction in the recruitment of total (5.5-fold less), CD8+ (10-fold less), and B cells (CD8-/B220+; 4.9-fold less) cells to the site of skin T_RM_ activation (Fig 5G–5I). Importantly, donor CFSE-labeled CD8+ and B cells (CD8-/B220+) cells were also significantly reduced in number (11- and 23-fold less, respectively) at the site of skin T_RM_ activation (Fig 5J and 5K). To exclude the possibility that post-septic environment influences the capacity of transferred memory CD8 T cells to respond to inflammation and/or cognate antigen stimulation recipient mice underwent sham or CLP surgery and 2 days later received splenocytes from LCMV-immune P14 chimera hosts (S7A Fig). Spleens were harvested after an additional 2 days and the memory P14 cells analyzed. Data presented in S7B–S7G Fig clearly showed that Ag-independent (bystander IFN-γ production in response to combination of inflammatory cytokines) and Ag-dependent (IFN-γ, TNF and IL-2 production in response to cognate antigen) responses of memory P14 CD8 T cells are normal, if not enhanced, even if those cells were exposed to post-septic environment. Thus, these data suggest the sepsis-induced reduction in recruitment of circulating effector cells into sites of T_RM_ activation is not solely due to numerical loss of these cells in circulation.

### Sepsis-induced lesion of vascular endothelium contributes to impaired skin CD8 T_RM_ responses

At this point, it was evident that recruitment of effector cells to the skin of septic hosts was greatly reduced—an observation that could not be exclusively attributed to impairments in skin T_RM_ Ag-dependent function to produce IFN-γ, the numerical loss of circulating cells that could be recruited to the inflamed peripheral site, or T cell intrinsic factor(s) that might prevent T cells exposed to sepsis or post-septic environment to follow inflammatory cues. Therefore, we focused on the contribution of T cell-extrinsic factors for the observed lesion in T_RM_-mediated responses.

**Fig 6:**
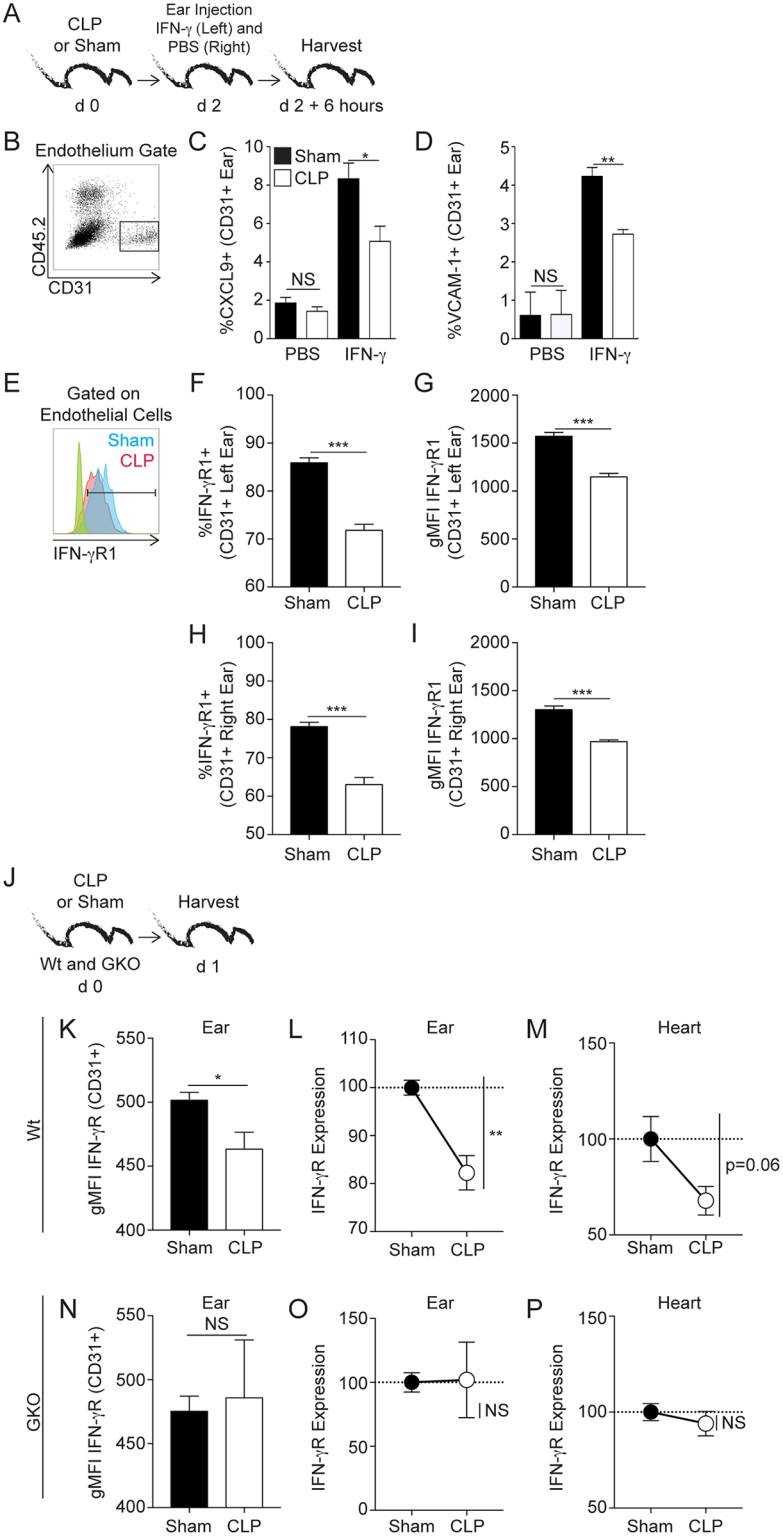
Sepsis impairs the capacity of endothelial cells to express molecules that promote IFN-γ mediated bystander recruitment. (A) Experimental Design. Naive C57Bl/6 mice underwent CLP or sham surgery. After 2 days, the mice received intradermal injections of 5μg recombinant IFN-γ (total volume 50μL) in the left ear and 50μL of PBS in the right ear. Ears were harvested 6 hours later and vascular endothelial cells analyzed. (B) Representative gating of skin endothelial cells (CD31+CD45.2-). (C) Frequency of endothelial cells producing CXCL9 or (D) VCAM-1 after PBS or IFN-γ exposure. (E) Representative histogram of skin endothelial cell IFN-γR1 expression, with FMO staining controls in green and orange colors representing sham and CLP, respectively. Frequency and gMFI of IFN-γR1 on endothelial cells from the left (F,G) or right (H, I) ear of VacV-immune mice two days after surgery. (J) Experimental design. Wildtype (Wt) or IFN-γ KO hosts (GKO) received CLP or sham surgery and 1 day later tissues of interest were harvested. (K) gMFI of IFN-γR1 on ear vascular endothelium of Wt hosts after sham or CLP surgery. IFN-γR1 expression on ear (L) or heart (M) vascular endothelium in Wt CLP host normalized to Wt sham hosts using gMFI values obtained from individual mice. Mean gMFI from Sham group is used as 100. (N) gMFI of IFN-γR1 expression on ear vascular endothelium of GKO hosts after sham or CLP surgery. IFN-γR1 expression on ear (O) or heart (P) vascular endothelium in GKO CLP host normalized to GKO sham hosts using gMFI values obtained from individual mice. Mean gMFI from Sham group is used as 100. Data are representative of two independent experiments with 3–5 mice per group per experiment. * = p<0.05; ** = p<0.01; *** = p<0.001. Error bars represent the standard error of the mean.

Decoration of the vascular endothelium with chemokines and adhesion molecules permits entry of other recirculating lymphocyte lineages into the site of T_RM_ activation [18]. As administration of exogenous IFN-γ into barrier tissues can recapitulate T_RM_-anamnestic responses (e.g., vascular endothelium upregulation of adhesion molecules and chemokines that promote bystander activation), we first examined the extent to which sepsis affects the capacity of vascular endothelial cells to respond to exogenous IFN-γ [18]. Naïve mice underwent CLP or sham surgery and 2 days later received IFN-γ or PBS in left and right ears, respectively, by i.d. injection (Fig 6A). While vascular endothelial cells (CD45.2- CD31+) from sham-treated mice had increased expression of CXCL9 and VCAM-1 after IFN-γ stimulation compared to endothelial cells in the contralateral ear exposed to PBS, we noted a significant reduction in CXCL9 and VCAM-1 expression on vascular endothelial cells from similarly treated mice post CLP (Fig 6B–6D). One potential explanation for this reduced CXCL9 and VCAM-1 expression on vascular endothelial cells in the post-septic environment was reduced expression of IFN-γR1 (Fig 6E). Importantly, the reduced IFN-γR1 expression on the endothelial cells was observed in both left and right ears suggesting a systemic change in skin vascular endothelium receptor expression during the immunoparalysis phase of sepsis (Fig 6F–6I).

To examine potential mechanisms driving the reduced IFN-γR1 expression on vascular endothelial cells early after sepsis induction new groups of sham and CLP mice were generated and the kinetics of IFN-γ secretion in the plasma were determined during the first 72 hours post-surgery (S8A Fig). CLP induces rapid and transient IFN-γ production (S8B Fig) suggesting that sepsis-induced IFN-γ during cytokine storm might lead to downregulation of IFN-γR1 expression as previously defined [42,43]. To test this, IFN-γ sufficient (wild type) and IFN-γ-deficient (GKO) mice underwent sham or CLP surgery and the expression of IFN-γR1 on CD31+ CD45.2- ear and heart endothelial cells assessed (Fig 6J). Importantly, sepsis-induced downregulation of IFN-γR1 on endothelial cells was not observed (Fig 6K–6P) in GKO mice strongly suggesting the contribution of sepsis-induced IFN-γ in IFN-γR1 downregulation.

**Fig 7:**
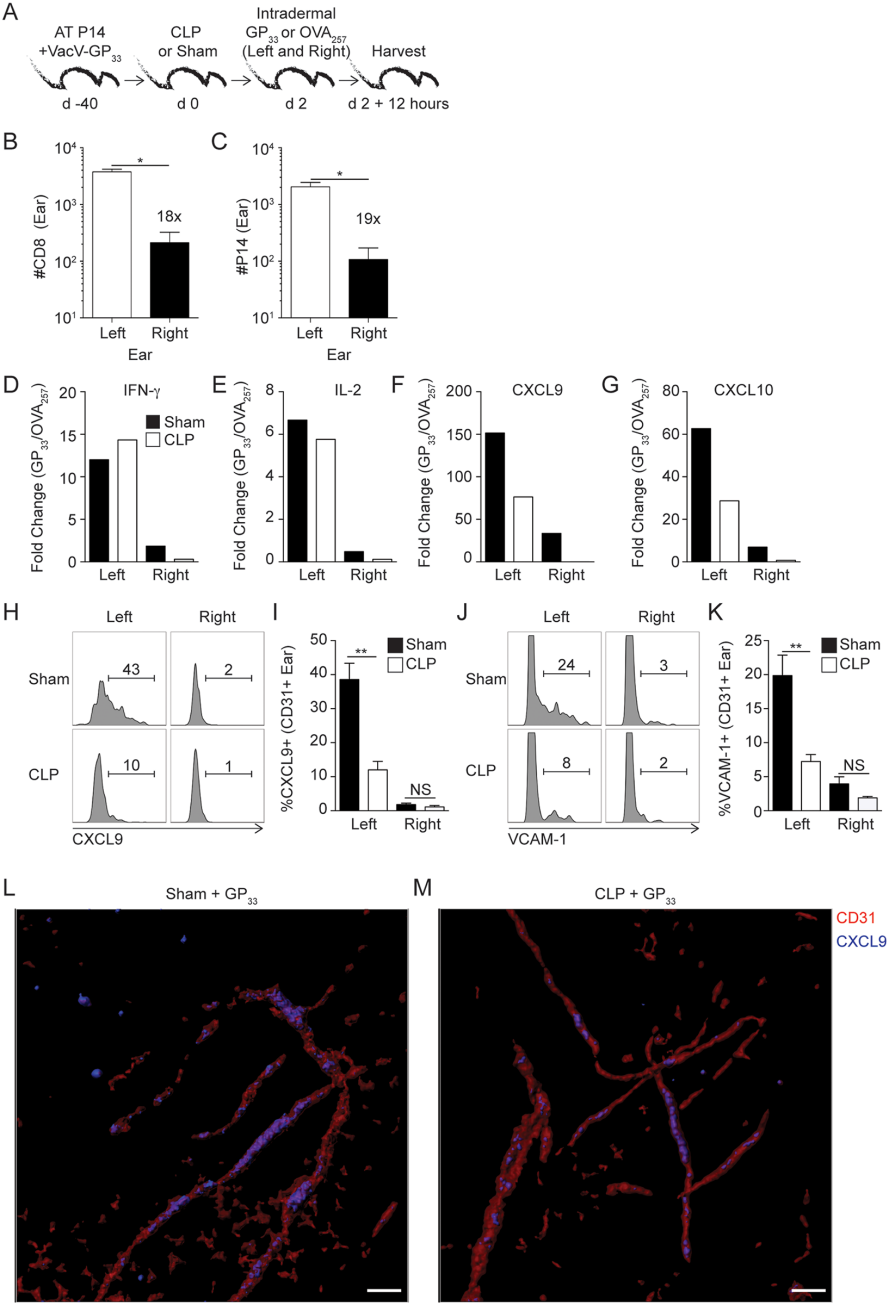
Sepsis impairs endothelial cells capacity to upregulate molecules that promote bystander recruitment in response to skin T_RM_ activation. (A) Experimental Design. 8x10^3^ naïve P14 CD8 T cells (Thy1.1) were adoptively transferred into C57Bl/6 recipient hosts (Thy1.2) followed by VacV-GP_33_ infection of the left ear. After 40 days mice underwent CLP or sham surgery. One group of mice received intradermal injection of 50μg of GP_33_ while second group of mice were injected with 50μg OVA_257_ peptide in both ears (left ear—primary and right ear—distal VacV infection sites) 2 days later. After 12 hours, mice received CD45.2 mAb injection and the left and right ears were harvested after 3 minutes. (B) Number of CD8 and (C) P14 skin T_RM_ in left and right ears on day 2 after sham surgery. (D-G) Whole ear RT-PCR screening of fold change increase (gene expression from mice that received GP_33_ peptide compared to OVA_257_ peptide injection) in the indicated gene expression of left and right ears. Representative gating and frequency of CXCL9 (H) or VCAM-1 (J) expression on endothelial cells in sham and CLP hosts after *in vivo* GP_33_ peptide stimulation. Frequency of CXCL9 (I) or VCAM-1 (K) expression on endothelial cells in left and right ears of sham or CLP mice. (L-M) Representative immunofluorescence images of epidermal ear mounts from Sham (L) or CLP (M) mice. Volume rendered isosurfacing of CD31 (red) and co-localization channel of CD31/CXCL9 (blue) were generated using the isosurfacing and co-localization features respectively in Imaris software. Data are representative of two independent experiments with 3–5 mice per group per experiment. NS = not significant; * = p<0.05; ** = p<0.01. Error bars represent the standard error of the mean or (D-G) mean values of group.

We continued our analysis of the effect of sepsis on vascular endothelial cells by evaluating their responsiveness to T_RM_-derived IFN-γ, which more accurately models an infection situation. T_RM_ (both total and Ag-specific P14 CD8 T cells) were significantly increased in the left ear (primary site of VacV-GP_33_-infection) compared to the uninfected contralateral right ear 30 days after infection (Fig 7B) [22,34]. This allowed us to determine the impact of T_RM_-derived IFN-γ in left and right ears by comparing the expression of endothelial cell-derived factors that promote bystander immune cell recruitment. VacV-GP_33_-immune mice underwent CLP or sham surgery before *in vivo* stimulation with OVA_257_ (irrelevant peptide) or GP_33_ (relevant peptide) in both ears in separate mice 2 days later. qRT-PCR was performed on the ear tissue after additional 12 hours (Fig 7A—model). In agreement with data presented before, sepsis did not affect the fold change in gene expression of T_RM_ effector cytokines, IFN-γ and IL-2, in the left ear (Fig 7D and 7E). The observed fold change increase was more evident in the left ear—a site where skin T_RM_ were present at a much higher number than the contralateral right ear. There was, though, a substantial reduction in mRNA expression of the CXCR3 ligands CXCL9 and CXCL10 in the left ear, which likely contribute to the reduction in T_RM_-mediated bystander immune cell recruitment in CLP-treated mice (Fig 7F and 7G) [18,19]. These data suggest the possibility that sepsis leads to sub-optimal production of CXCR3 ligands that facilitate bystander effector cells recruitment upon skin T_RM_ activation despite sufficient IFN-γ production from skin T_RM_. In a subsequent experiment, we confirmed the qRT-PCR data by examining the expression of CXCL9 and VCAM-1 protein on the vascular endothelium after skin T_RM_ activation. Upregulation of these molecules on endothelial cells was primarily relegated to the site of VacV infection (Fig 7H–7K), but was significantly reduced in the CLP-treated mice. Finally, examining this process topologically using immunofluorescence we confirmed that CXCL9 co-localization of CD31+ vascular endothelium of the ear is diminished in the septic host upon T_RM_ activation (Fig 7L and 7M). Lastly, we tested the ability of endothelial cells to respond to T_RM_-derived IFN-γ after homologous VacV-GP_33_ infection (S9A Fig). Consistent with data above, there was significantly reduced expression of CXCL9 and VCAM-1 on the endothelial cells in tissue harvested from CLP-treated mice compared to the sham counterparts (S9B and S9C Fig). Taken together, sepsis-induced IFN-γ modulates the expression of IFN-γR1 on the vascular endothelial cells preventing proper recognition of T_RM_-derived IFN-γ to trigger upregulation of chemokines and/or adhesion molecules required for proper bystander recruitment of effector cells to the inflamed periphery.

### Exogenous CXCL9/10 administration at time of skin T_RM_ activation improves effector cell recruitment in septic hosts

**Fig 8:**
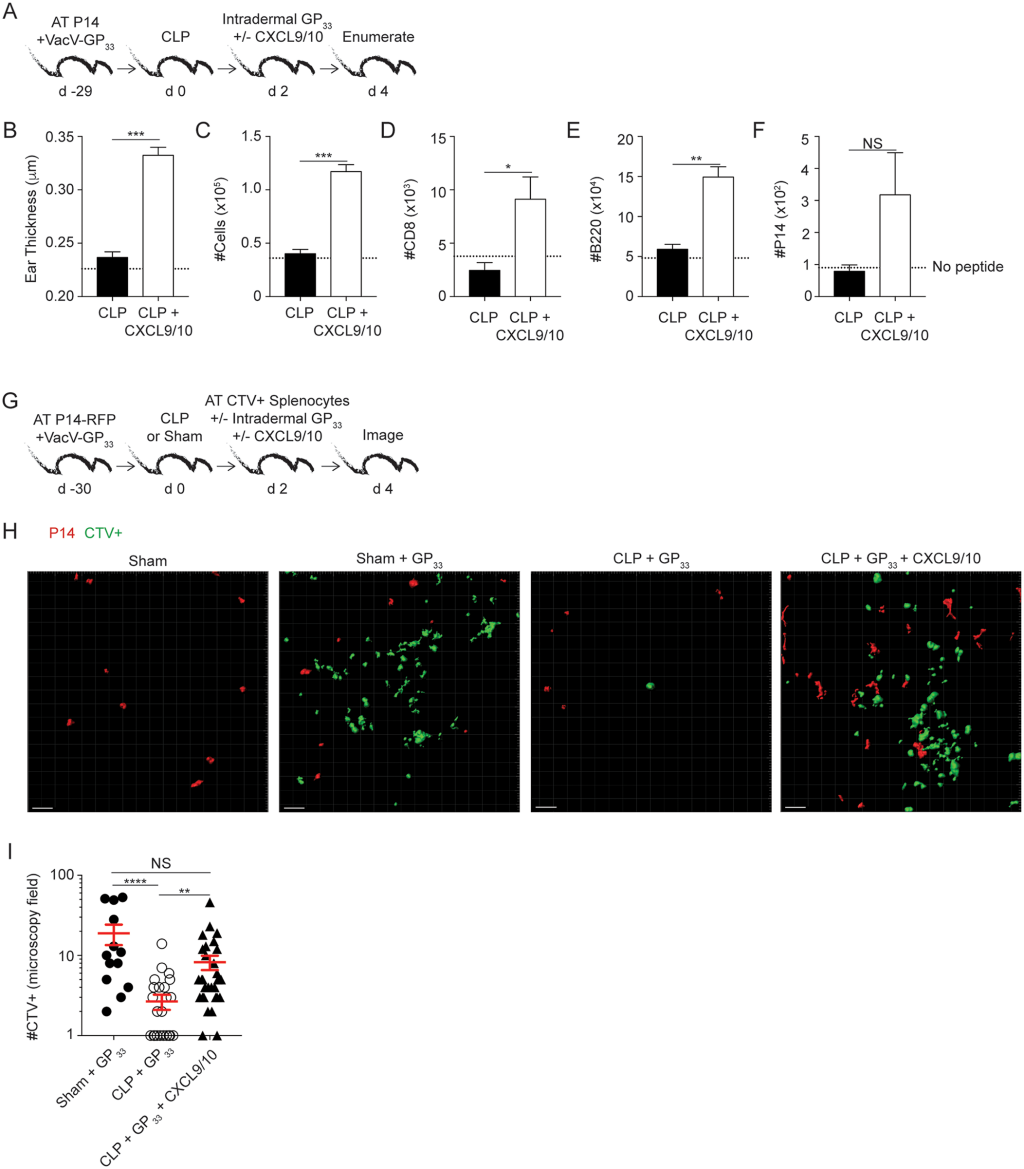
Exogenous CXCL9/10 administration at time of skin T_RM_ activation improves effector cell recruitment in septic hosts. (A) Experimental design. Two days after CLP surgery left ears of VacV-GP_33_ immune P14 chimera mice were treated *in vivo* with GP_33_ peptide with or without CXCL9/10 treatment. (B) Thickness of left ear pinna after *in vivo* peptide stimulation with or without CXCL9/10. Total number of cells (C) or number of CD8+ CD45.2- (D), B220+ CD45.2- (E), and P14 CD45.2- (F) cells in the ear after skin T_RM_ activation in the presence or absence of CXCL9/10. Dashed line represent number of cells in control CLP hosts that did not receive *in vivo* peptide injection. (G) Experimental design. Two days after CLP surgery VacV-GP_33_ immune P14-RFP chimera mice were adoptively transferred with CTV labeled splenocytes from an LM-OVA immunized host before intradermal GP33 injection in the presence or absence of exogenous CXCL9/10. Two days later, intravital microscopy was performed to image cells in the ear *in situ*. (H) Host and *in vivo* stimulation conditions are listed above a representative image of intravital microscopy of the ear. Red cells are P14-RFP cells residing in the ear and green cells are CTV labeled splenocytes from LM-OVA donor animals. (I) Number of CTV cells per microscopic field in the ear of sham and CLP hosts with specified *in vivo* stimulation conditions. Statistical analysis (B-F) student t-test. (I) 1-way ANOVA corrected for multiple comparisons using Dunn’s test. Data representative from at least two experiments with 3–7 mice per group. NS = not significant. * = p<0.05; ** = p<0.01; *** = p<0.001; **** = p<0.0001. Error bars represent the standard error of the mean.

To address the exciting possibility that exogenous administration of CXCR3 chemokines are sufficient to improve effector cell recruitment to the site of T_RM_ activation VacV-GP_33_-immune P14 chimera mice received CLP surgery 2 days before intradermal injection of GP_33_ peptide in the presence or absence of CXCL9/10 chemokines (Fig 8A). Dramatic increase in ear thickness and number of cells (total and/or effector T and B cell populations) was observed in the ear of CLP mice that received CXCR3 chemokines (Fig 8B–8F). We next visualized the T_RM_-mediated recruitment of effector cells into the periphery by adoptively transferring naïve P14-RFP TCR-Tg CD8 T cells into congenic B6 mice before VacV-GP_33_ infection of the ear. At memory time point surgery was performed and 2 days later sham and CLP mice received adoptive transfer of CTV-labelled splenocytes obtained from *Listeria monocytogenes* immune donors (Fig 8G). Immediately after the transfer, ears containing P14-RFP memory cells were injected with GP_33_ peptide in the presence or absence of CXCL9/10 chemokines and the number of RFP+ or CTV+ cells visualized with 2-photon intravital microscopy 2 days later (Fig 8G). Corroborating data shown previously, the calculated number of RFP+ P14 cells per microscopy field of the ear was not different between sham and CLP mice (10.5 +/- 3.7 vs 11.1 +/- 2.5, respectively). However, sepsis prevented efficient recruitment of CTV+ cells upon T_RM_ peptide activation that was restored after exogenous CXCL9/10 administration (Fig 8H and 8I) suggesting a potential way to correct sepsis-induced impairments in effector cell recruitment to the site of localized infection/inflammation.

### Sepsis diminishes protection against localized VacV re-challenge

**Fig 9:**
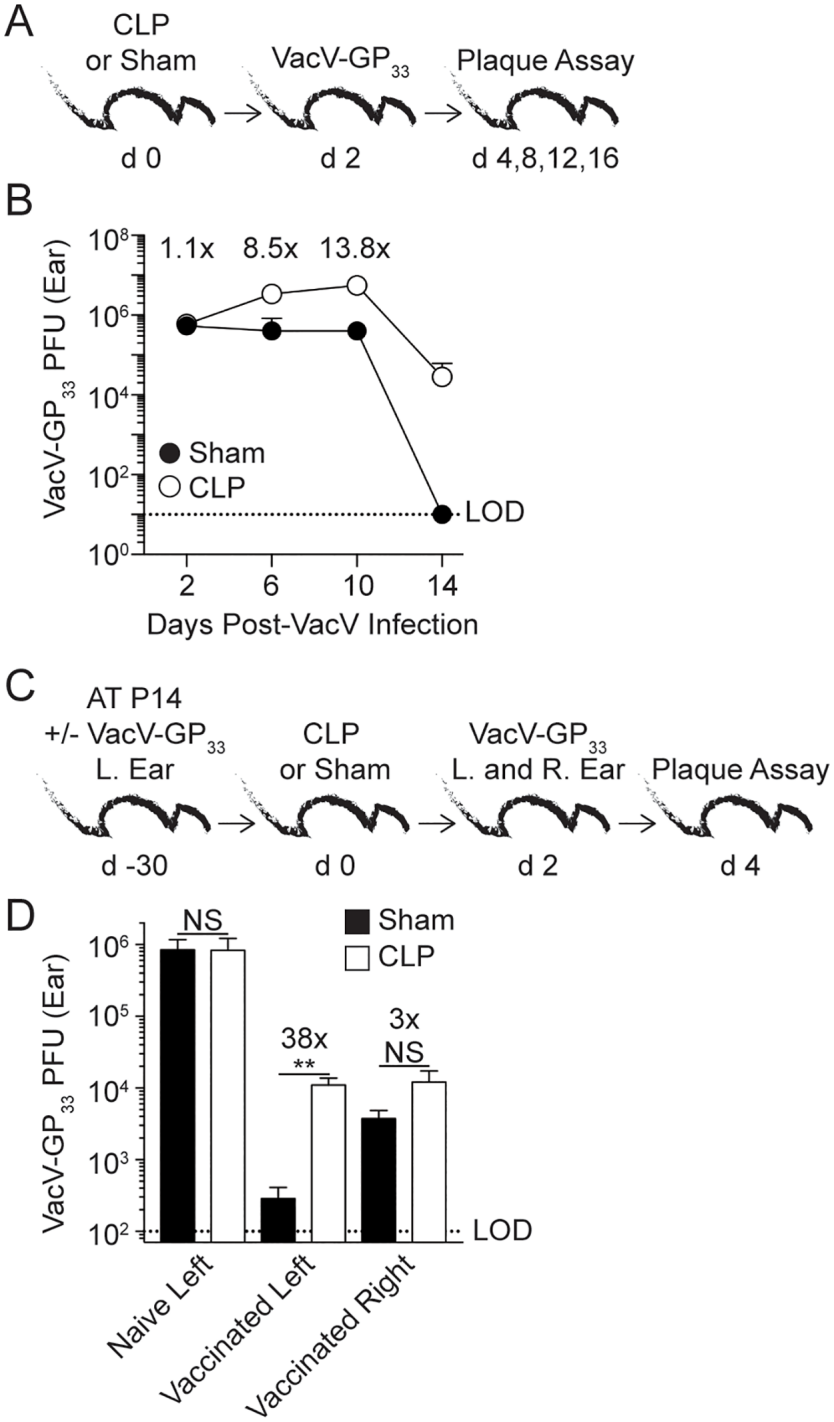
Protection to VacV re-challenge is diminished in VacV-immune mice after sepsis induction. (A) Experimental design. Naive hosts underwent CLP or sham surgery and two days later received VacV infection in the left ear and viral titer was determined at various times post infection. (B) Plaque forming units (PFU) of the infected left ear of CLP or sham hosts after infection. Numbers above data points represent the fold change in PFUs. Dashed line represents the LOD for the plaque assay. (C) Experimental design. 10^4^ naive P14 CD8 T cells (Thy1.1) were adoptively transferred into C57Bl/6 recipients (Thy1.2) that remained naïve (non-vaccinated) or received VacV-GP_33_ infection of the left ear (vaccinated). After 30 days mice underwent CLP or sham surgery. The mice were then infected in left and right ears with VacV-GP_33_ 2 days later, followed by plaque assay two days after infection. (D) PFU of the infected left ear of non-vaccinated and left and right ear of vaccinated animals 2 days after VacV-GP_33_ infection. Data are representative of two similar experiments with 3–8 mice per group. Numbers above bars show fold change in viral titer between groups. LOD = level of detection. NS = not significant; ** = p<0.01. Error bars represent the standard error of the mean.

Due to the critical interplay between T_RM_ and T_CIRCM_ in providing optimal protection upon pathogen re-encounter we next examined the impact of sepsis on the T_RM_-mediated protection after VacV-GP_33_ re-challenge. However, the capacity of memory CD8 T cells to provide protection is always addressed in comparison to naïve, non-immunized host. The fact that sepsis increases susceptibility of naïve host to various types of bacterial or viral infections [8,9,11,44] could complicate the interpretation of the results in which the contribution of T_RM_ cell-mediated protection is determined. To precisely define the time after VacV-infection when viral load in the infected ear is not markedly influenced by sepsis, naïve mice underwent sham or CLP surgery 2 days before VacV infection of the left ear (Fig 9A). Although sepsis clearly increased susceptibility to VacV infection similar viral load is observed 2 days post infection in CLP and sham hosts (Fig 9B) giving us the opportunity to use this model to address the role of sepsis in shaping T_RM_ cell-mediated protection.

Naïve mice were infected in the left ear to generate T_RM_ at the site of primary infection. Infected and naïve groups of mice were challenged with VacV-GP_33_ in left and right ears 2 days after sham or CLP surgery (Fig 9C). Two days after infection, naïve hosts showed high viral burden with no appreciable difference in viral titers after sepsis induction (Fig 9D), just as determined in Fig 9A and 9B. Interestingly, viral burden was ~38-fold higher in the left ears of CLP- compared to sham-treated immune mice strongly suggesting that sepsis has the capacity to impair T_RM_ cell-mediated protection to localized virus re-challenge (Fig 9D).

To confirm the contribution of T_RM_ in homologous anti-VacV protection that was not dependent on the other arms of adaptive immunity (e.g. anti-VacV antibodies generated after vaccination) viral burden was also examined in right ears that had similar contributions of anti-VacV immune responses from the periphery except significantly reduced numbers of local T_RM_. Importantly, viral clearance was reduced in the contralateral ears (compared to the left ears) in vaccinated sham hosts, suggesting the number of skin T_RM_ indeed influences viral clearance upon homologous VacV infection [22,24]. However, there was only a 3-fold increase in viral burden in the contralateral right ears of vaccinated CLP-treated mice, suggesting the sepsis influence on non T_RM_-mediated arms of immunity is less pronounced (Fig 9D). Finally, septic hosts had similar viral burden in both ears despite dramatically different number of skin T_RM_ strongly suggesting CD8 T_RM_ function is diminished upon sepsis induction. Thus, sepsis reduces protection against VacV re-challenge by preventing efficient recognition of T_RM_–dependent ‘alarming’ signals at the site of localized infection.

## Discussion

Sepsis is a robust immune response against a systemic infection that can lead to widespread host tissue damage or death [45]. The early phase of sepsis is characterized by a cytokine storm resulting from the production of a variety of pro- and anti-inflammatory cytokines that contribute to the pathophysiological responses leading to early death of septic patients [46]. After resolution of the cytokine storm, the host enters a period of immunosuppression hallmarked by robust lymphocyte apoptosis and enhanced susceptibility to secondary infections and latent viral reactivation (e.g., HSV) [6,47]. We and others have used the CLP model of sepsis because of its similarities to clinical manifestations of sepsis [48], and we have previously described how CLP-induced sepsis reduces the quantity and quality of naïve and memory CD4 and CD8 T cells [8,9,12,15,49]. Moreover, we recently detailed how the consequences of sepsis on the number and function of dendritic cells contribute to the observed impairments in primary T cell responses [14]. Thus, it is likely that intrinsic and extrinsic lesions contribute to the sub-optimal T cell-dependent responses in septic hosts [15].

The ability to develop and sustain memory CD8 T cells after immunization or infection is a hallmark of the adaptive immune response and a basis for protective vaccination against infectious disease. Memory CD8 T cells possess several important features that distinguish them from naïve CD8 T cells. They are increased in numbers providing better coverage and a more rapid response during pathogen re-encounter. Memory CD8 T cells have experienced cell-intrinsic “programming” changes that allow for rapid expression of effector cytokines, chemokines, and cytotoxic molecules. Finally, and most relevant to the work described herein, memory CD8 T cells can establish residence in both lymphoid (T_CIRCM_) and non-lymphoid tissues (T_RM_). By taking advantage of a simple *in vivo* labeling procedure, various quantitative and qualitative parameters of CD8 T_CIRCM_ and T_RM_ can be interrogated. Although CD8 T cells that are i.v.-negative are broadly considered CD8 T_RM_, some memory CD8 T cells could have transient residence within previously infected tissues. Thus, in addition to CD103 expression, parabiosis could be used to rigorously define ‘true’ tissue residence in the i.v.-negative CD8 T cell compartment.

The evaluation of both CD8 T_CIRCM_ and T_RM_ cell populations from the same mice allowed us to confirm previously reported sepsis-induced alterations in CD8 T_CIRCM_ cells while extending our investigation to include the understudied CD8 T_RM_ population. Sepsis-induced impairments in the number and Ag-dependent functions were evident in “i.v.-positive” T_CIRCM_ to a greater extent than “i.v.-negative” CD103+ T_RM_ counterparts in multiple barrier tissues. Interestingly, one report observed a numerical loss of memory CD8 T cells in the blood and SLO (spleen) but no numerical changes were noted in nonlymphoid tissues, including the lungs [50]. An important limitation of this previous report was the absence of intravascular staining to distinguish between Ag-specific memory CD8 T cells in circulation and tissue parenchyma [31,32]. While it remains unclear which factors are responsible for the numerical and functional maintenance of T_RM_ in septic hosts, we can posit a few explanations. CD8 T_RM_ are phenotypically distinct from their T_CIRCM_ counterparts, which could potentially alter the susceptibility of these populations to the various factors driving sepsis-induced lymphopenia [16]. Additionally, the local environment in which CD8 T_RM_ and T_CIRCM_ reside may vary widely during the hyperinflammatory phase of sepsis. It stands to reason that T_CIRCM_ located in blood and SLO may have greater exposure to pro- and anti- inflammatory cytokines produced during the cytokine storm than cells separated from the circulation within the parenchyma of barrier tissues, leading to the differences in sepsis-induced numerical loss.

Another important question to be answered is how CD8 T_RM_ in various barrier tissues are numerically and/or functionally altered long after the initial septic event. While we observed similar numbers of CD8 T_RM_ in all barrier tissues examined (skin, lung, and gut), it is possible that the long-term numerical maintenance of CD8 T_RM_ after sepsis induction depends on the tissue of residence. For example, skin CD8 T_RM_ are numerically stable over time, suggesting this population might maintain optimal numbers long-term because sepsis does not initially impair the number of CD8 T_RM_. In contrast, evidence exists that lung CD8 T_RM_ do not maintain themselves numerically and require constant replacement and seeding of the lung with their Ag-specific T_CIRCM_ counterparts. It is believed this is required because of the high rate of homeostatic turnover of lung CD8 T_RM_, which we also report here [51,52]. Thus, our ‘snapshot’ analysis of lung CD8 T_RM_ may not accurately reflect the long-term consequences of sepsis on this population. It is important to reiterate that barrier tissues have vastly different local environments and while we show similarity between all examined tissues we acknowledge it is possible CD8 T_RM_ throughout the body may not behave identically after sepsis induction [53]. Future experiments will be designed to address this important question.

Vascular endothelial cell dysfunction has been widely described in sepsis and results in low blood pressure in septic patients [54]. Early after sepsis induction, the vascular endothelium responds to the cytokine storm by increasing expression of adhesion molecules (e.g. VCAM-1) to promote interaction with innate immune cells which produce factors, including oxygen radicals and elastase, that lead to dysfunction or death of vascular endothelium [55,56,57,58]. Interestingly, the production of compounds that affect the normal function of the vascular endothelium correlate with outcome during sepsis [59,60,61]. Therefore, it stands to reason the degree of vascular endothelium dysfunction might be altered based on sepsis severity. Our observed impairments of vascular endothelium to respond to T_RM_-derived IFN-γ suggest an additional T cell extrinsic factor contributing to sub-optimal memory CD8 T cell responses in septic hosts [15]. This finding is important given recent therapeutic strategies meant to restore number and/or function of memory CD8 T cells in septic patients, including cytokine therapy (IL-7 or IL-15) and checkpoint blockade (anti-PD-L1) [10,62,63]. This report further supports the notion that merely focusing on fixing T cell impairments after sepsis induction may not fully restore CD8 T cell-mediated responses [14,15].

In summary, we showed that sepsis had minimal impact on number and Ag-dependent ‘sensing’ and ‘alarming’ function of CD8 T_RM_ in a variety of barrier tissues. However, the ability of skin T_RM_ to recruit other circulating immune effector cells to the site of skin T_RM_ activation after local Ag deposit or homologous infection was significantly decreased in septic hosts. We determined IFN-γ produced shortly after sepsis induction altered IFN-γR1 expression and function of vascular endothelium leading to impairments in VCAM-1 and CXCL9 expression upon exposure to exogenous or T_RM_-derived IFN-γ. Importantly, recombinant CXCL9 and CXCL10 application at the site of T_RM_ activation was sufficient to restore the described lesion in effector cell recruitment to the skin. Chemokine therapy in animal models has proven beneficial in promoting protective immune responses in situations where normal host immune responses are insufficient to mediate protection. Specifically, chemokine therapy has been used in tissue-specific immunization strategies against HSV2 [64] and recruiting anti-tumor immune responses to the site of tumor development [65,66]. Thus, this finding suggests CXCL9 and CXCL10 administration at the site of secondary skin infections could improve the localized immune responses of septic patients as a supplement to conventional antibiotic interventions. These data also build on our previous findings to suggest sepsis-induced suppression of memory CD8 T cell immunity is due to both T cell-intrinsic and -extrinsic lesions, which together contribute to the increased susceptibility of sepsis survivors to previously-encountered infections.

## Materials and methods

### Ethics statement

Experimental procedures using mice were approved by University of Iowa Animal Care and Use Committee under ACURF protocol numbers 1312217 and 6121915. The experiments performed followed Office of Laboratory Animal Welfare guidelines and PHS Policy on Humane Care and Use of Laboratory Animals.

### Mice, pathogens and memory CD8 T cell generation

Inbred C57Bl/6 (B6; Thy1.2/1.2) and outbred Swiss Webster mice were purchased from the National Cancer Institute (Frederick, MD) and maintained in the animal facilities at the University of Iowa at the appropriate biosafety level. IFN-γ knockout (GKO) mice and P14 T cell receptor-transgenic (TCR-Tg) mice (Thy1.1/1.1) were bred and maintained at the University of Iowa (Iowa City, IA). B6.CAG.MRFP1-mice were obtained from Jackson labs and crossed with B6.P14-Thy1.1 mice at the University of Iowa to yield RFP-P14 Thy1.1 B6 mice. LCMV-Armstrong (2x10^5^ PFU) was injected i.p. Recombinant ActA-deficient *Listeria monocytogenes* expressing OVA (LM-OVA) was injected i.v. (1x10^7^). For influenza infection, mice were anesthetized with isoflurane and recombinant PR8-GP_33_ (4x10^4^ TCID50 provided by Dr. Steven Varga, University of Iowa) was administered i.n. VacV-GP_33_ or VacV WR infection of the skin was performed by applying 5x10^6^ PFU in 5 μl saline to the center of the ear pinna and then poking it 30 times with a 27-gauge needle [34]. VacV-GP_33_ viral titers were quantified using plaque assay on BSC-40 cells, as described previously [67].

5-10x10^3^ naïve P14 cells obtained from peripheral blood of naïve P14 mice (Thy1.1) were adoptively transferred into B6 recipients (Thy1.2) i.v., followed by infection with a pathogen expressing the GP_33-41_ epitope of LCMV.

### Cell isolation

Peripheral blood (PBL) was collected by retro-orbital bleeding. Ears, small intestine, and lungs were treated with collagenase type II (Worthington, 100 U/mL) in RPMI 1640 supplemented with 5% fetal calf serum (FCS) and shaken at 450 RPM for 30–90 min at 37°C. Single cell suspension was prepared by mashing tissues through a 70 μm cell strainer (Falcon) with the plunger of a 1 mL syringe (BD Biosciences). Samples were centrifuged and re-suspended in RPMI. Single-cell suspensions from spleen, lymph nodes and heart were generated after mashing tissue through 70 μm cell strainer without enzymatic digestion.

### Flow cytometry, peptides and cytokine detection

Flow cytometry data were acquired on a FACSCanto (BD Biosciences, San Diego, CA) and analyzed with FlowJo software (Tree Star, Ashland, OR). To determine expression of cell surface proteins, mAb were incubated at 4°C for 20–30 min and cells were fixed using Cytofix/Cytoperm Solution (BD Biosciences) and, in some instances followed by mAb incubation to detect intracellular proteins. The following mAb clones were used: CD8 (53–6.7; eBioscience), Thy1.1 (HIS51; eBioscience), B220 (RA3-6B2; eBioscience), CD45.2 (104; eBioscience), IFN-γ (XMG1.2; eBioscience), TNF-α (MP6-XT22, eBioscience), IL-2 (JES6-5H4, eBioscience), CD103 (2E7; eBioscience), CD31 (390, Biolegend), CXCL9 (MIG-2F5.5), and VCAM-1 (429; eBioscience), CXCR3 (CXCR3-173, eBioscience), CD62L (MEL-14, eBioscience), CD27 (LG.7F9, eBioscience), KLRG-1 (2F1, eBioscience), CD127 (A7R34, eBioscience), CD122 (5H4, eBioscience), CD69 (H1.2F3, eBioscience), T-bet (eBio410, eBioscience) Eomesodermin (Dan11mag, eBioscience), Granzyme B (MHGB04, Invitrogen), CD107a (1D4B, BD Pharmingen). IFN-γ receptor was detected using CD119-Biotin (2E2; eBioscience) and Streptavidin-PE (eBioscience) after acid stripping, as described previously [68]. Plasma concentrations of IFN-γ were determined using Multiplex Immunoassay (ProcartaPlex; Affymetrix by eBioscience) on a Bio-Rad BioPlex, as performed previously [69].

Intravascular stain protocol to distinguish circulatory from resident cells: two minutes after APC-conjugated CD45.2 mAb was injected i.v. into mice, peripheral blood was collected by retro-orbital bleeding and used as a positive control with >99% memory P14 cells routinely labeled with CD45.2 mAb [32]. After an additional minute, mice were euthanized and organs of interest were harvested and processed for flow cytometry.

Intracellular cytokine staining: for *in vitro* bystander responses, splenocytes were incubated for 4 hours at 37°C with recombinant IL-12 and IL-18, or IL-12 and TNF, or IL-18 and IFN-β (10 ng/mL each) (R&D Systems). Cells were incubated for 1 additional hour in the presence of Brefeldin A (BFA) before surface and intracellular IFNγ staining. For Ag-dependent CD8 T cell responses splenocytes were incubated with 200nM GP_33-41_ peptide at 37°C for 5 hours in the presence of BFA before surface and intracellular cytokine staining as described [70].

GP_33-41_ (KAVYNFATM) and OVA_257-264_ (SIINFEKL) peptides were synthesized by Bio-Synthesis (Louisville, TX).

Apoptosis was evaluated using Vybrant FAM Caspase-3/7 Assay Kit (Invitrogen) according to manufacturer’s protocol.

### *In vivo* peptide stimulation of skin T_RM_ and CXCL9/10 administration

Skin T_RM_ in VacV-immunized hosts were activated *in situ* with 50μg transdermal injection of indicated peptides at the immunization site as described [34]. Single cells suspension from the ears is obtained 6 hours later and stained for surface and intracellular antigens/cytokines in the absence of BFA. When stated, 3μg of CXCL9 and CXCL10 (Peprotech) was added to the site of peptide injection as previously described [64].

### Memory CD8 T cell migration assay

LCMV-immune mice splenocytes were resuspended in migration medium (5x10^6^ cells per mL of 0.1% FCS supplemented 1xHBSS) and plated in 96 well transwell plate with polystyrene membrane and 5μm pores (Corning) as described [40]. Transwell insert was removed and migration medium supplemented with 1,000ng/mL of CXCL9 or CXCL10 (Peprotech) added to receiving plate. With transwell insert returned to receiving plate 100 μl of splenocytes suspension was added to transwell and incubated for 90 minutes at 37°C. Migrated cells were collected in the receiving plate and stained as described previously. P14 and Ag-experienced (CD8α^lo^CD11a^hi^) CD8 T cell populations were enumerated using flow cytometric ‘Count’ in the receiving plate using FlowJo software.

### CFSE and CTV cell labeling

Splenocytes (10^7^/mL) from LCMV-immune mice were labeled with CarboxyFluorescein diacetate Succinimidyl Ester (CFSE; eBioscience) by incubating the cells at room temperature for 15 minutes with 5μM CFSE. The labeled cells were incubated for 5 minutes with 1mL FCS on ice to remove any free CFSE, and washed three times with RPMI 1640 supplemented with 10% FCS prior to adoptive transfer by i.v. injection, as we performed previously [70].

Splenocytes isolated from LM-OVA immune mice were labeled with CellTrace Violet (CTV, Life Technologies) by incubating the cells at 37°C for 20 minutes with 10mM CTV. The labeled cells were then incubated for 5 minutes with 1mL FCS on ice to remove any free CTV, and washed three times with RPMI 1640 supplemented with 10% FCS prior to adoptive transfer by i.v. injection, as performed previously [71].

### Ear thickness

An electronic digital micrometer (Marathon) 0–1” range was placed on the center of the ear pinna to record thickness, as reported previously [34].

### Quantitative RT-PCR

Total RNA was reverse-transcribed using a QuantiTech Reverse Transcription Kit (Qiagen). The resulting cDNA was analyzed for expression of different genes by quantitative PCR using SYBR Advantage qPCR pre-mix (Clontech) on an ABI 7300 Real Time PCR System (Applied Biosystems). Relative gene expression levels in each sample were normalized to beta-actin (ACTB). Primers used in quantitative RT-PCR were:

IFN-γ: forward-GCGTCATTGAATCACACCTG and reverse-TGAGCTCATTGAATGCTTGG; IL-2: forward-AACCTGAAACTCCCCAGGAT and reverse-CGCAGAGGTCCAAGTTCATC; CXCL9: forward-TCGGACTTCACTCCAACACAG and reverse-AGGGTTCCTCGAACTCCACA; CXCL10: forward-CCACGTGTTGAGATCATTGCC and reverse-GAGGCTCTCTGCTGTCCATC; and ACTB: forward-CGGTTCCGATGCCCTGAGGCTCTT and reverse-CGTCACACTTCATGATGGAATTGA.

### Cecal ligation and puncture (CLP) model of sepsis induction

Mice were anesthetized with ketamine/xylazine (University of Iowa, Office of Animal Resources), the abdomen was shaved and disinfected with Betadine (Purdue Products), and a midline incision was made. The distal third of the cecum was ligated with Perma-Hand Silk (Ethicon), punctured once using a 25-gauge needle, and a small amount of fecal matter extruded. The cecum was returned to abdomen, the peritoneum was closed with 641G Perma-Hand Silk (Ethicon), and skin sealed using surgical Vetbond (3M). Following surgery, 1 mL PBS was administered s.c. to provide post-surgery fluid resuscitation. Bupivacaine (Hospira) was administered at the incision site, and flunixin meglumine (Phoenix) was administered for postoperative analgesia. This procedure created a septic state characterized by loss of appetite and body weight, ruffled hair, shivering, diarrhea, and/or periorbital exudates with 0–10% mortality rate, similar to our previous reports [8,9,12,14]. Sham mice underwent identical surgery excluding cecal ligation and puncture.

### Intravital 2-photon microscopy

RFP P14 donor mice were bled and 2-5x10^3^ naïve RFP P14s were adoptively transferred to mice by i.v. injection one day prior to infection. 5x10^6^ PFU of VacV-GP_33_ was administered to the ear as previously described and mice underwent CLP or Sham surgery at 30–40 days post infection. 1.5x10^7^ CTV labeled splenocytes from LM-OVA infected donors were transferred 48 hours post-surgery into CLP or Sham mice. Ears were stimulated with GP_33_ peptide and imaged for RFP P14 and CTV cells with live intravital 2-photon imaging 48 hours post peptide injection.

All images were acquired on a Leica SP8 Microscope (Leica) using a 25x / 0.95 NA water immersion objective with coverslip correction with 1.25x zoom. High resolution (512 x 512) stacks of 20–35 xy sections sampled with 3μm z spacing were acquired at an acquisition rate of 40 frames/second to provide image volumes of 170/388 x 170/388 x 60–105μm. Mice were anesthetized with Ketamine/Zylazine (87.5/12.5 mg/Kg). The VacV immune ears that contained RFP P14 T_RM_ cells of live mice were positioned on the microscope base in a continuously heated enclosed chamber (Leica). A custom suction tissue window apparatus (VueBio) was placed on the Ear with 20-25mm Hg of negative pressure to gently immobilize the tissue against a fixed coverslip. Images were acquired only in regions containing RFP+ P14s, irrespective of the presence or absence of CTV+ splenocytes. Images were sequentially excited with a tunable Mai Tai HP Sapphire laser (Spectra Physics) with excitation of CTV at 800nm and emission 420-490nm and RFP P14s with excitation at 1080nm and emission at 570-700nm. Images were acquired at a rate of 1 image per 60–90 seconds due to the delay in laser tuning. Sequences of image stacks were transformed into volume-rendered, 3D images and 4D time-lapse movies with Imaris Version 8.1 (Bitplane).

### Whole epidermal mounts immunofluorescence and confocal microscopy

VacV-GP_33_ immune mice underwent sham or CLP surgery. Whole epidermal ear mounts were processed from isolated ears, as described [72]. Briefly, ears from non-stimulated or GP_33_-peptide stimulated sham or CLP mice were collected 24 hours after stimulation, treated with Nair (Church and Dwight Co.) for 5 minutes to remove hair, affixed to slides with super glue and incubated in 10mM EDTA in PBS for 2 hours at 37°C. The dermis was physically removed from the epidermis and the epidermal sheets were fixed in 4% paraformaldehyde for 2 hours at 4°C. Tissues were rinsed in TBS and blocked with CAS-Block (Life Science Technologies) and stained with CD3-Alexa594, CD31-Alexa488, and CXCL9- Alexa647 (Biolegend) at 4°C for 12–16 hrs. Sections were rinsed in TBS and mounted on slides with ProLongGold (Invitrogen). Images were acquired with sequential imaging with excitation of 488nm and emission 500-550nm for Alexa488, excitation at 561 and emission at 600–650 for Alexa594 and excitation at 633 and emission at 650-720nm for Alexa647. Sequences of image stacks were transformed into volume-rendered, 3D images with Imaris Version 8.4 (Bitplane).

### Statistical analysis

Unless stated otherwise data were analyzed using Prism6 software (GraphPad) using two-tailed Student t-test with a confidence interval of >95% to determine significance (*p ≤ 0.05, **p ≤ 0.01, ***p ≤ 0.001, ****p ≤ 0.0001 and N.S. as not significant) data are presented as standard error of the mean.

## Supporting information

S1 Fig Intravascular labeling distinguishes CD8 T_CIRCM_ and skin T_RM_. (A) Experimental design. C57Bl/6 (Thy1.2) mice received adoptive transfer of 5 × 10^3^ naïve P14 (Thy1.1) cells followed by VacV-GP_33_ infection of the left ear. Mice received intravascular injection of CD45.2 mAb 30 days later, followed by tissue harvesting 3 minutes later. (B) Left column: representative gating of memory P14 cells within blood, left and right ear of VacV-immune mice. Middle column: representative histogram of CD45.2 mAb labeling of memory P14 cells within the blood (T_CIRCM_) and left ear (T_RM_) of VacV-immune mice. Right column: representative histogram of CD103 expression on P14 T_CIRCM_ and skin T_RM_ populations. (C) Experimental Design. C57Bl6 (Thy1.2) mice received adoptive transfer of 5 × 10^3^ naïve P14 (Thy1.1) cells followed by intraperitoneal LCMV-Armstrong infection. Mice received intravascular injection of CD45.2 mAb 160 days later, followed by tissue harvesting after another 3 minutes. (D) Representative gate of memory P14 in the left ear of LCMV-immune mice. Data are representative of two independent experiments with 2–4 mice per group per experiment.
(TIF)

S2 Fig Kinetics of CD8 T cell death after sepsis induction. (A) Experimental design. VacV-immune hosts received sham or CLP surgery and CD8 T cells from peripheral blood were analyzed at indicated hours after surgery. (B) Number of Ag-experienced CD8 T cells distinguished using the surrogate activation marker (CD8α^lo^CD11a^hi^) at time after surgery. Dashed line represents numerical average of Ag-experienced CD8 T cells 6 hours after sham surgery. (C) Representative histograms of activated caspase 3/7 in Ag-experienced CD8 T cells after sham or CLP surgery at indicated time points after surgery. (D) Experimental design. At a memory time point VacV-GP_33_ immune P14 chimera mice underwent sham or CLP surgery and four days later tissues of interest were harvested. (E) Number of P14 T_CIRCM_ in the spleen and (F) Number of P14 skin T_RM_ (CD45.2-CD103+) four days after surgery. Data are representative of two experiments with at least 4 mice per group. NS = not significant, * = p<0.05. Error bars represent the standard error of the mean.
(TIF)

S3 Fig Sepsis reduces number of P14 and total CD8 T_CIRCM_ to a greater extent than lung T_RM_ in influenza-immune mice. (A) Experimental design. C57Bl/6 (Thy1.2) mice received 8 × 10^3^ naïve P14 (Thy1.1) cells followed by intranasal PR8-GP_33_ infection. Mice underwent CLP or sham surgery 35 days later. The mice then received an intravascular injection of CD45.2 mAb 2 days later, followed by tissue harvested after another 3 minutes. (B) Representative histogram of CD45.2 mAb labeling of lung P14 cells in PR8-GP_33_ immune mice. Ratio of CD45.2+:CD45.2- lung P14 cells is shown. (C) Summary data of lung P14 cells ratio of CD45.2+:CD45.2- in CLP or sham flu-immune mice. (D) Number of CD45.2+ and (E) CD45.2- CD103+ P14 cells within lung. (F) Number of splenic P14 cells two days after surgery. (G) Experimental design. C57Bl/6 (Thy1.2) mice received intranasal infection of PR8-GP_33_ and 38 days later mice underwent CLP or sham surgery. The mice received an intravascular injection of CD45.2 mAb 2 days later, and tissues were harvested after 3 minutes. (H) Gating strategy of total CD8 T cells. (I) Representative histogram of CD45.2 mAb labeling of lung CD8 T cells in PR8-GP_33_ immune mice that underwent CLP or sham surgery. Ratio of CD45.2+:CD45.2- CD8 T cells. (J) Ratio of CD45.2+:CD45.2- lung CD8 T cells in CLP or sham flu-immune mice summary data. (K) Number of CD45.2+ or CD45.2- lung CD8 T cells in CLP or sham flu-immune mice. (L) Representative histogram of activated caspase-3/7 of CD45.2- and CD45.2+ lung CD8 T cells. (M) Frequency of activated caspase-3/7 of CD45.2- lung CD8 T cells and (N) CD45.2+ lung CD8 T cells. Data representative of three independent experiments with 3–5 mice per group per experiment. NS = not significant; * = p<0.05; **** = p<0.0001. Error bars represent the standard error of the mean.
(TIF)

S4 Fig Sepsis reduces the number P14 T_CIRCM_ to a greater extent than lung and gut T_RM_ in LCMV-immune mice. (A) Experimental Design. 7x10^3^ naïve P14 cells (Thy1.1) were adoptively transferred into C57Bl/6 recipients (Thy1.2) followed by intraperitoneal LCMV-Armstrong infection. After 30 days mice underwent CLP or sham surgery. Two days later mice received intravascular injection of CD45.2 mAb, and tissues were harvested three minutes later and cells enumerated. (B) Representative histogram of CD45.2 mAb labeling in small intestine and lung memory P14 cells. Representative ratio of CD45.2+:CD45.2- P14 cells is shown in CLP and sham mice. (C) Summary data of CD45.2+:CD45.2- ratio of memory P14 cells in small intestine and (D) lung. (E) Number of CD45.2- and CD45.2+ lung P14 cells in CLP and sham mice. Data are representative of three independent experiments with 3–5 mice per group per experiment. NS = not significant; * = p<0.05; ** = p<0.01; **** = p<0.0001. Error bars represent the standard error of the mean.
(TIF)

S5 Fig Intradermal antigen injection stimulates IFN-γ production by skin T_RM_ and T_CIRCM_ *in vivo*. (A) Experimental design. 6 hours after intradermal injection of peptide (+GP_33_ group) or non-manipulated (-GP_33_ group) ears and spleens from VacV-GP_33_ immune P14 chimera mice were harvested and spiked *ex vivo* with CFSE labeled splenocytes from an LCMV immune P14 chimera donor mouse. (B) Gating strategy of CFSE- host P14 cells and CFSE+ 'sensor' P14 cells. Representative histograms of IFN-γ production of host and ‘sensor’ P14 skin T_RM_ (C) and spleen T_CIRCM_ (D). Data from 3 mice per group.
(TIF)

S6 Fig Sepsis does not change the phenotype and cytolytic potential of skin resident memory CD8 T cells. (A) Experimental design. Representative histograms of indicated (B) surface markers, (C) transcription factors, and (D) IFN-γR1 expression on CD45.2-/CD103+ P14 skin T_RM_ from sham or CLP hosts. (E) Experimental design. VacV-GP_33_ immune P14 chimera mice underwent sham or CLP surgery and two days later skin cells were obtained. To facilitate antigen recognition skin cells were mixed with 2x10^6^ of naïve congenic splenocytes during *in vitro* GP_33_ stimulation. (F) Representative histogram of Granzyme B and (G) CD107a expression of P14 T_RM_ after GP_33_ peptide stimulation. (H) Summary data of CD107a production of skin T_RM_ from sham or CLP hosts in the presence or absence of GP_33_ peptide. Data representative from 3 mice per group. NS = not significant. Error bars represent the standard error of the mean.
(TIF)

S7 Fig Memory CD8 T cells exposed to post-acute septic environment are not impaired in Ag-independent or -dependent responses. (A) Experimental design. Naive mice underwent CLP or sham surgery and two days later received adoptive transfer of splenocytes from LCMV-immune P14 chimera hosts. After additional two days spleens were harvested and the capacity of P14 cells to respond to inflammation and/or cognate antigen-stimulation analyzed. (B-D) Ag-independent bystander IFN-γ production of memory P14 cells after exposure to indicated combinations of inflammatory cytokines. Production of (E) IFN-γ (F) TNF, and (G) IL-2 after *in vitro* stimulation with GP_33_ peptide. Data from at least 3 mice per group. NS = not significant. ** = p<0.01. Error bars represent the standard error of the mean.
(TIF)

S8 Fig Sepsis induces rapid and transient IFN-γ production. (A) Experimental design. Naive mice received CLP or sham surgery and plasma samples obtained at indicated hours after surgery. (B) Amount of IFN-γ in plasma samples of CLP or sham hosts. LOD of the assay was 0.9 pg/mL. Representative data from two similar experiment with 1–4 mice per group per time point. Error bars represent the standard error of the mean.
(TIF)

S9 Fig Sepsis impairs endothelial cells capacity to upregulate molecules that promote bystander recruitment in response to CD8 skin T_RM_ activation. (A) Experimental Design. 8 x 10^3^ naïve P14 CD8 T cells (Thy1.1) were adoptively transferred into C57Bl/6 recipient hosts (Thy1.2) followed by VacV-GP_33_ infection of the left ear. After 30 days mice underwent CLP or sham surgery and 2 days later mice received homologous VacV-GP_33_ infection in the same ear. Frequency of (B) CXCL9 and (C) VCAM-1 expression on endothelial cells of the left and right ears 2 days after secondary VacV-infection. Data are representative of two independent experiments with 3–5 mice per group per experiment. NS = not significant; ** = p<0.01. Error bars represent the standard error of the mean.
(TIF)
