# Supplementary figures and images for: Polymicrobial sepsis impairs bystander recruitment of effector cells to infected skin despite optimal sensing and alarming function of skin resident memory CD8 T cells
Source: PLoS Pathog. 2017 Sep 14;13(9):e1006569. doi: 10.1371/journal.ppat.1006569 (PMC5599054; doi:10.1371/journal.ppat.1006569)

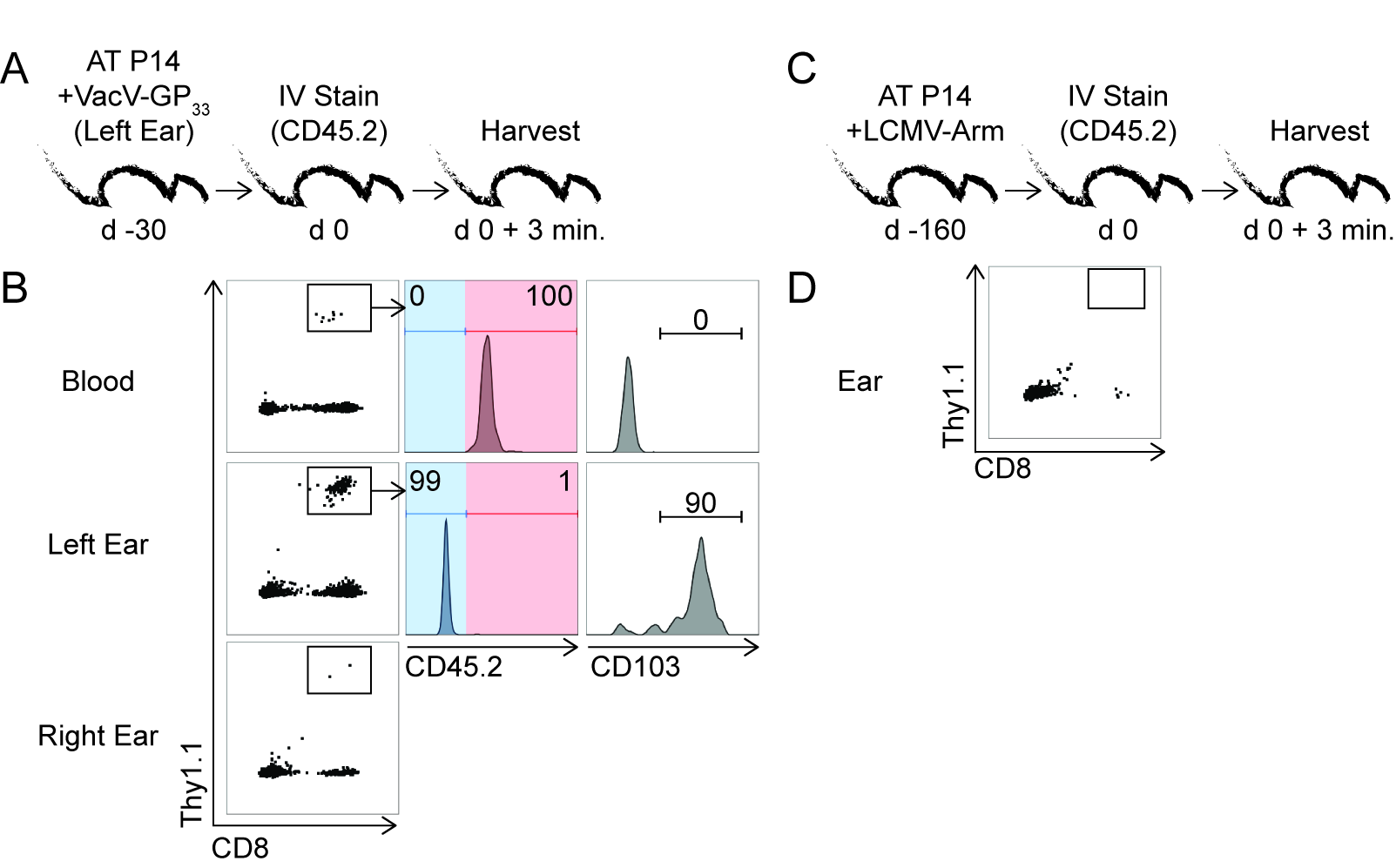

Supplement: S1 Fig — (A) Experimental design. C57Bl/6 (Thy1.2) mice received adoptive transfer of 5 × 10^3^ naïve P14 (Thy1.1) cells followed by VacV-GP33 infection of the left ear. Mice received intravascular injection of CD45.2 mAb 30 days later, followed by tissue harvesting 3 minutes later. (B) Left column: representative gating of memory P14 cells within blood, left and right ear of VacV-immune mice. Middle column: representative histogram of CD45.2 mAb labeling of memory P14 cells within the blood (TCIRCM) and left ear (TRM) of VacV-immune mice. Right column: representative histogram of CD103 expression on P14 TCIRCM and skin TRM populations. (C) Experimental Design. C57Bl6 (Thy1.2) mice received adoptive transfer of 5 × 10^3^ naïve P14 (Thy1.1) cells followed by intraperitoneal LCMV-Armstrong infection. Mice received intravascular injection of CD45.2 mAb 160 days later, followed by tissue harvesting after another 3 minutes. (D) Representative gate of memory P14 in the left ear of LCMV-immune mice. Data are representative of two independent experiments with 2–4 mice per group per experiment. (TIF) [file ppat.1006569.s001.tif]

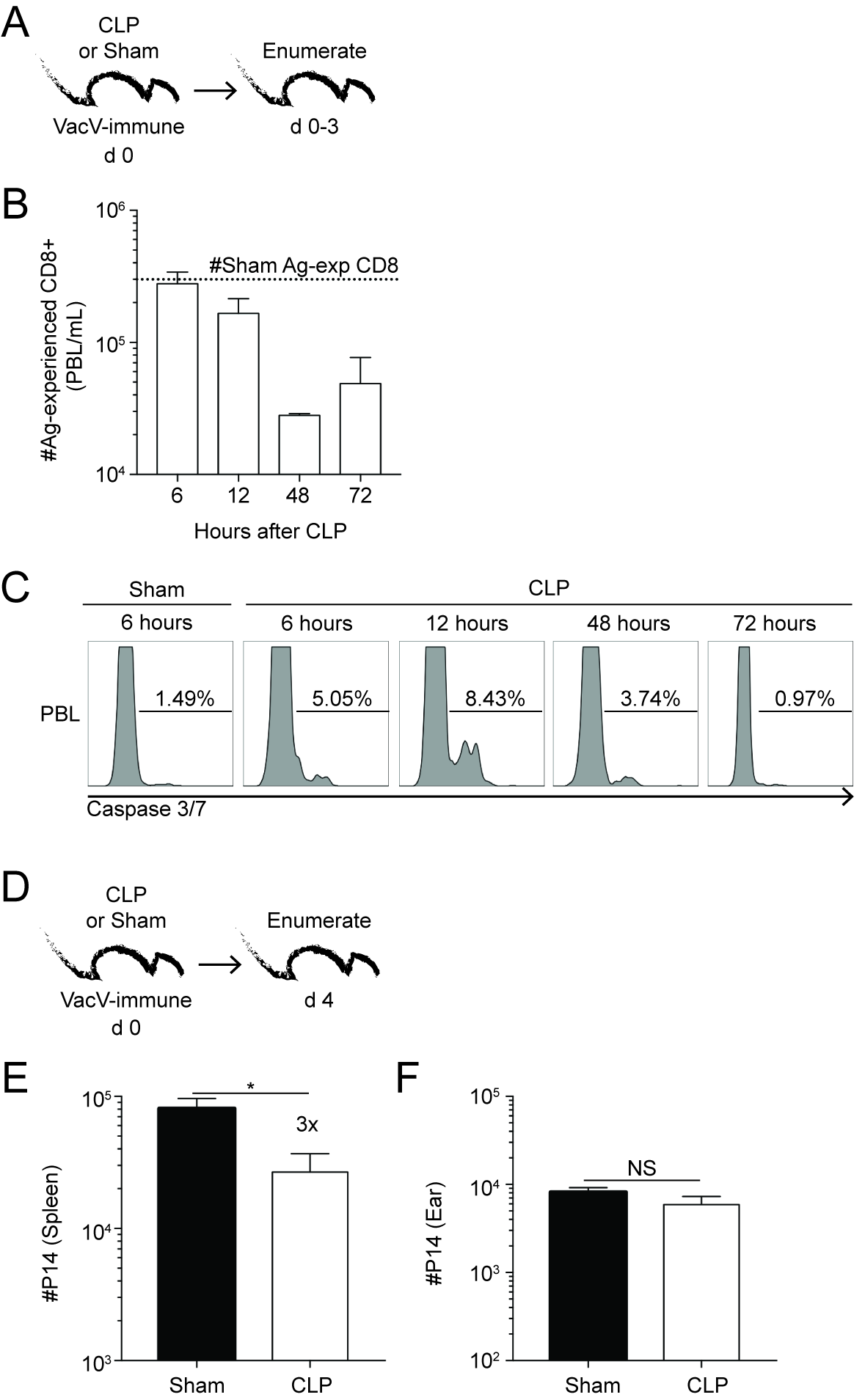

Supplement: S2 Fig — (A) Experimental design. VacV-immune hosts received sham or CLP surgery and CD8 T cells from peripheral blood were analyzed at indicated hours after surgery. (B) Number of Ag-experienced CD8 T cells distinguished using the surrogate activation marker (CD8αloCD11ahi) at time after surgery. Dashed line represents numerical average of Ag-experienced CD8 T cells 6 hours after sham surgery. (C) Representative histograms of activated caspase 3/7 in Ag-experienced CD8 T cells after sham or CLP surgery at indicated time points after surgery. (D) Experimental design. At a memory time point VacV-GP33 immune P14 chimera mice underwent sham or CLP surgery and four days later tissues of interest were harvested. (E) Number of P14 TCIRCM in the spleen and (F) Number of P14 skin TRM (CD45.2-CD103+) four days after surgery. Data are representative of two experiments with at least 4 mice per group. NS = not significant, * = p<0.05. Error bars represent the standard error of the mean. (TIF) [file ppat.1006569.s002.tif]

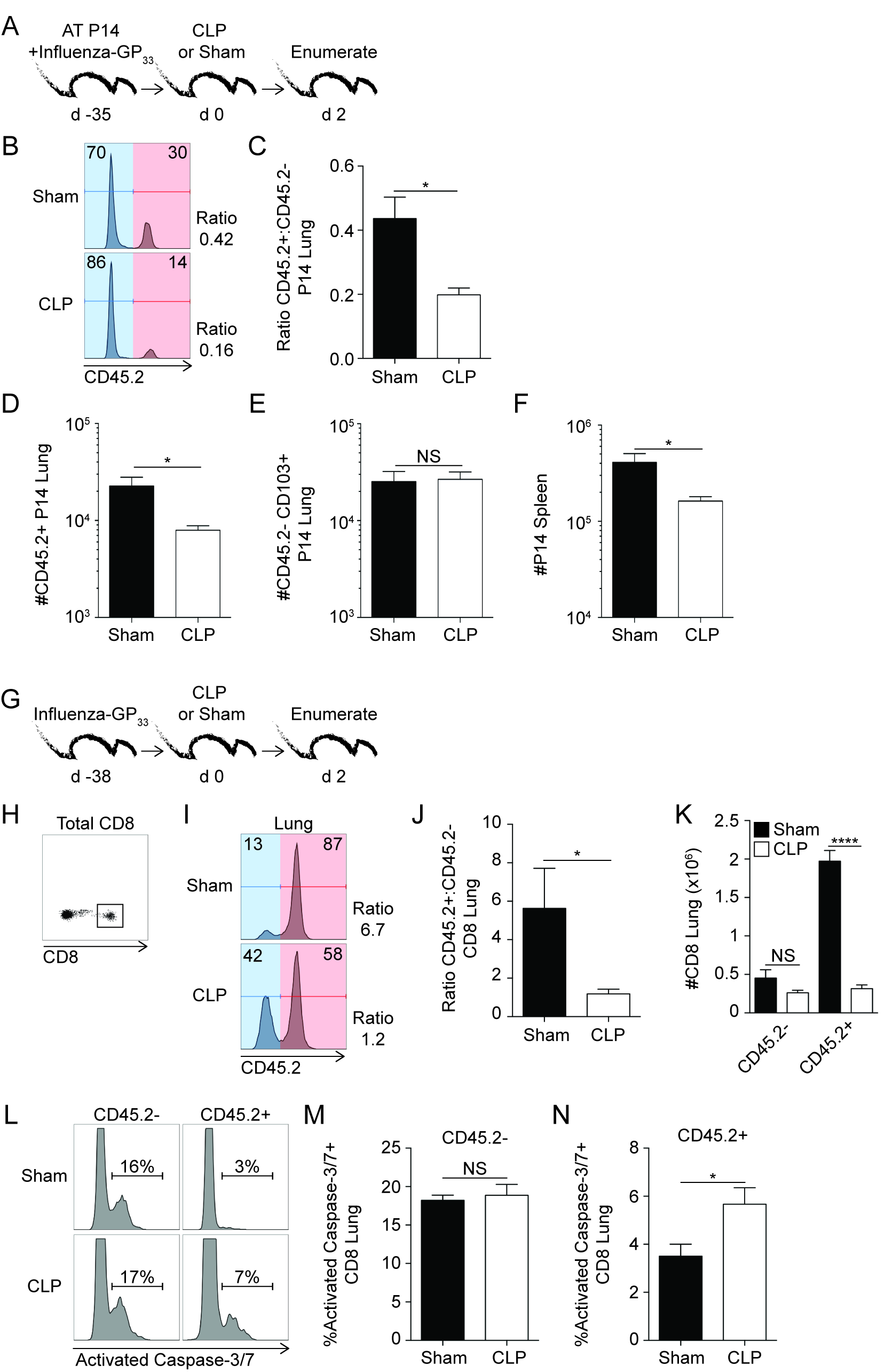

Supplement: S3 Fig — (A) Experimental design. C57Bl/6 (Thy1.2) mice received 8 × 10^3^ naïve P14 (Thy1.1) cells followed by intranasal PR8-GP33 infection. Mice underwent CLP or sham surgery 35 days later. The mice then received an intravascular injection of CD45.2 mAb 2 days later, followed by tissue harvested after another 3 minutes. (B) Representative histogram of CD45.2 mAb labeling of lung P14 cells in PR8-GP33 immune mice. Ratio of CD45.2+:CD45.2- lung P14 cells is shown. (C) Summary data of lung P14 cells ratio of CD45.2+:CD45.2- in CLP or sham flu-immune mice. (D) Number of CD45.2+ and (E) CD45.2- CD103+ P14 cells within lung. (F) Number of splenic P14 cells two days after surgery. (G) Experimental design. C57Bl/6 (Thy1.2) mice received intranasal infection of PR8-GP33 and 38 days later mice underwent CLP or sham surgery. The mice received an intravascular injection of CD45.2 mAb 2 days later, and tissues were harvested after 3 minutes. (H) Gating strategy of total CD8 T cells. (I) Representative histogram of CD45.2 mAb labeling of lung CD8 T cells in PR8-GP33 immune mice that underwent CLP or sham surgery. Ratio of CD45.2+:CD45.2- CD8 T cells. (J) Ratio of CD45.2+:CD45.2- lung CD8 T cells in CLP or sham flu-immune mice summary data. (K) Number of CD45.2+ or CD45.2- lung CD8 T cells in CLP or sham flu-immune mice. (L) Representative histogram of activated caspase-3/7 of CD45.2- and CD45.2+ lung CD8 T cells. (M) Frequency of activated caspase-3/7 of CD45.2- lung CD8 T cells and (N) CD45.2+ lung CD8 T cells. Data representative of three independent experiments with 3–5 mice per group per experiment. NS = not significant; * = p<0.05; **** = p<0.0001. Error bars represent the standard error of the mean. (TIF) [file ppat.1006569.s003.tif]

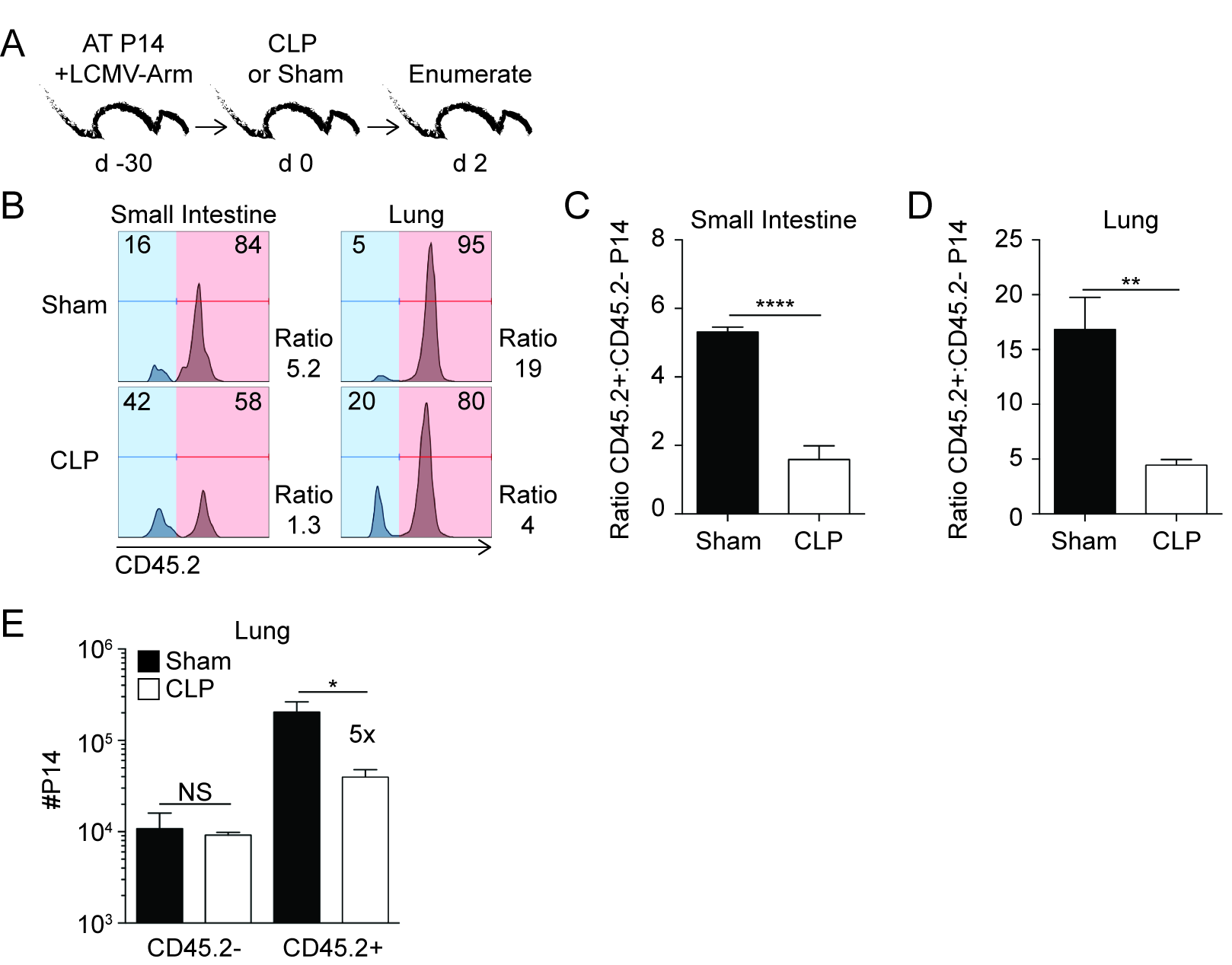

Supplement: S4 Fig — (A) Experimental Design. 7x10^3^ naïve P14 cells (Thy1.1) were adoptively transferred into C57Bl/6 recipients (Thy1.2) followed by intraperitoneal LCMV-Armstrong infection. After 30 days mice underwent CLP or sham surgery. Two days later mice received intravascular injection of CD45.2 mAb, and tissues were harvested three minutes later and cells enumerated. (B) Representative histogram of CD45.2 mAb labeling in small intestine and lung memory P14 cells. Representative ratio of CD45.2+:CD45.2- P14 cells is shown in CLP and sham mice. (C) Summary data of CD45.2+:CD45.2- ratio of memory P14 cells in small intestine and (D) lung. (E) Number of CD45.2- and CD45.2+ lung P14 cells in CLP and sham mice. Data are representative of three independent experiments with 3–5 mice per group per experiment. NS = not significant; * = p<0.05; ** = p<0.01; **** = p<0.0001. Error bars represent the standard error of the mean. (TIF) [file ppat.1006569.s004.tif]

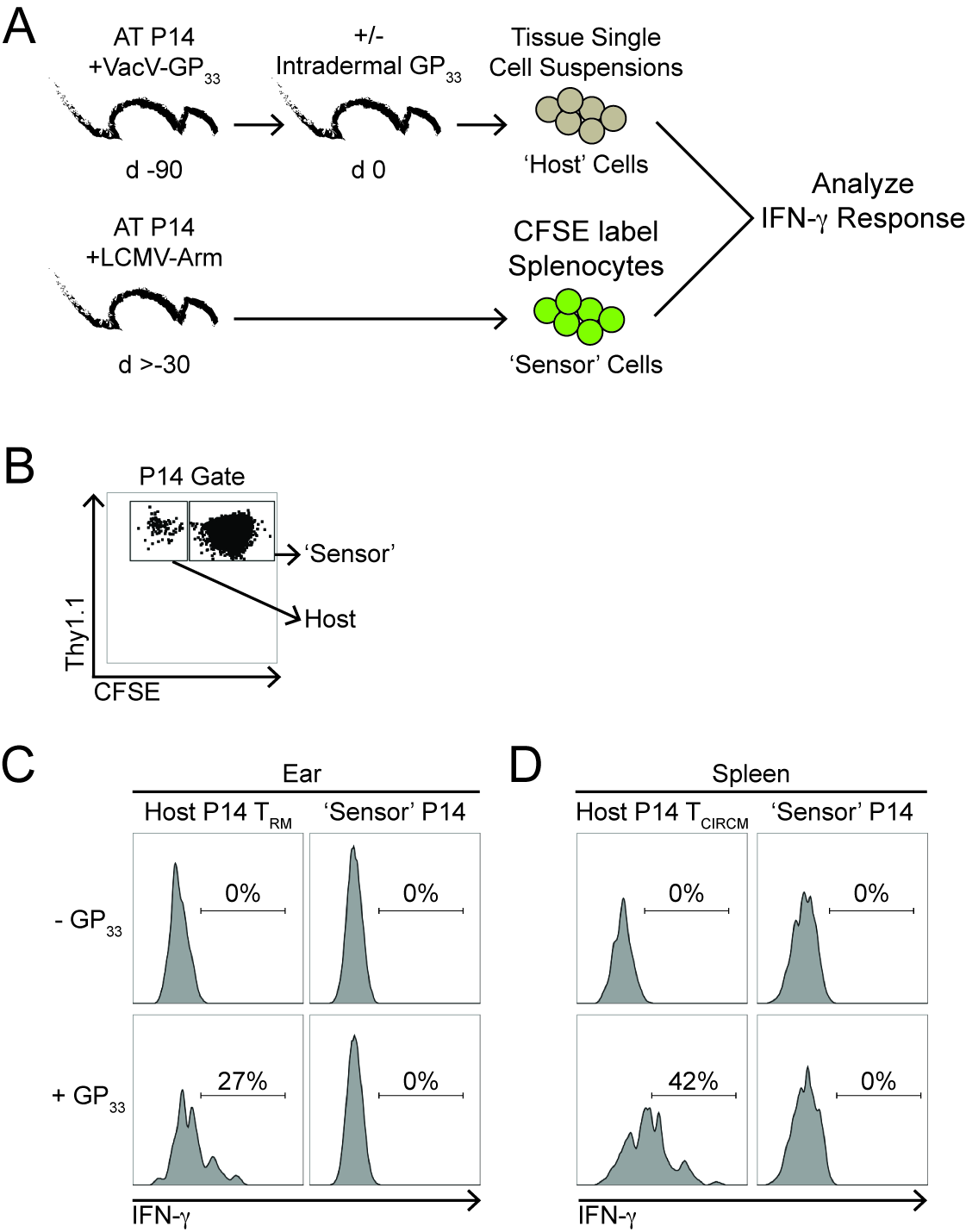

Supplement: S5 Fig — (A) Experimental design. 6 hours after intradermal injection of peptide (+GP33 group) or non-manipulated (-GP33 group) ears and spleens from VacV-GP33 immune P14 chimera mice were harvested and spiked ex vivo with CFSE labeled splenocytes from an LCMV immune P14 chimera donor mouse. (B) Gating strategy of CFSE- host P14 cells and CFSE+ 'sensor' P14 cells. Representative histograms of IFN-γ production of host and ‘sensor’ P14 skin TRM (C) and spleen TCIRCM (D). Data from 3 mice per group. (TIF) [file ppat.1006569.s005.tif]

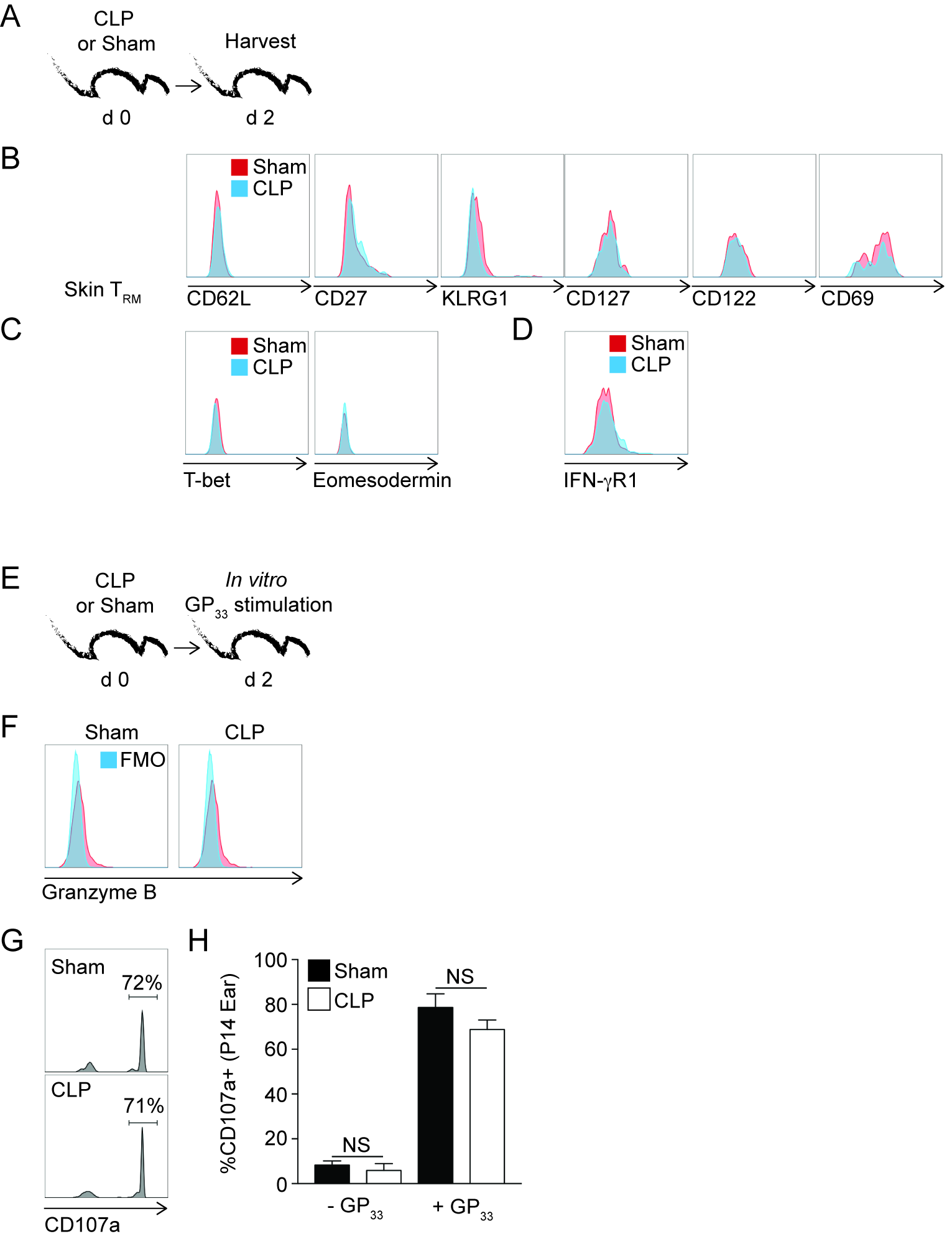

Supplement: S6 Fig — (A) Experimental design. Representative histograms of indicated (B) surface markers, (C) transcription factors, and (D) IFN-γR1 expression on CD45.2-/CD103+ P14 skin TRM from sham or CLP hosts. (E) Experimental design. VacV-GP33 immune P14 chimera mice underwent sham or CLP surgery and two days later skin cells were obtained. To facilitate antigen recognition skin cells were mixed with 2x10^6^ of naïve congenic splenocytes during in vitro GP33 stimulation. (F) Representative histogram of Granzyme B and (G) CD107a expression of P14 TRM after GP33 peptide stimulation. (H) Summary data of CD107a production of skin TRM from sham or CLP hosts in the presence or absence of GP33 peptide. Data representative from 3 mice per group. NS = not significant. Error bars represent the standard error of the mean. (TIF) [file ppat.1006569.s006.tif]

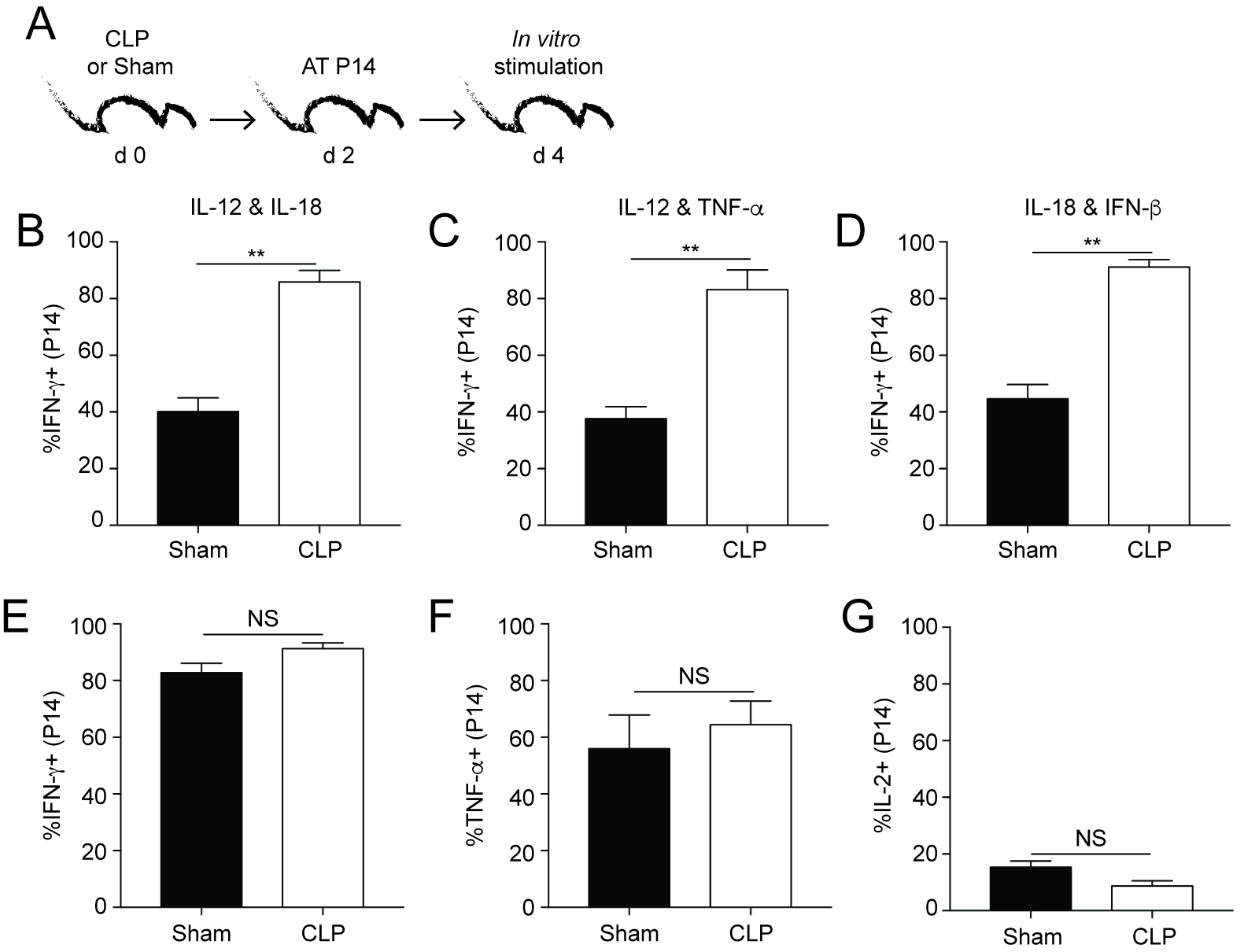

Supplement: S7 Fig — (A) Experimental design. Naive mice underwent CLP or sham surgery and two days later received adoptive transfer of splenocytes from LCMV-immune P14 chimera hosts. After additional two days spleens were harvested and the capacity of P14 cells to respond to inflammation and/or cognate antigen-stimulation analyzed. (B-D) Ag-independent bystander IFN-γ production of memory P14 cells after exposure to indicated combinations of inflammatory cytokines. Production of (E) IFN-γ (F) TNF, and (G) IL-2 after in vitro stimulation with GP33 peptide. Data from at least 3 mice per group. NS = not significant. ** = p<0.01. Error bars represent the standard error of the mean. (TIF) [file ppat.1006569.s007.tif]

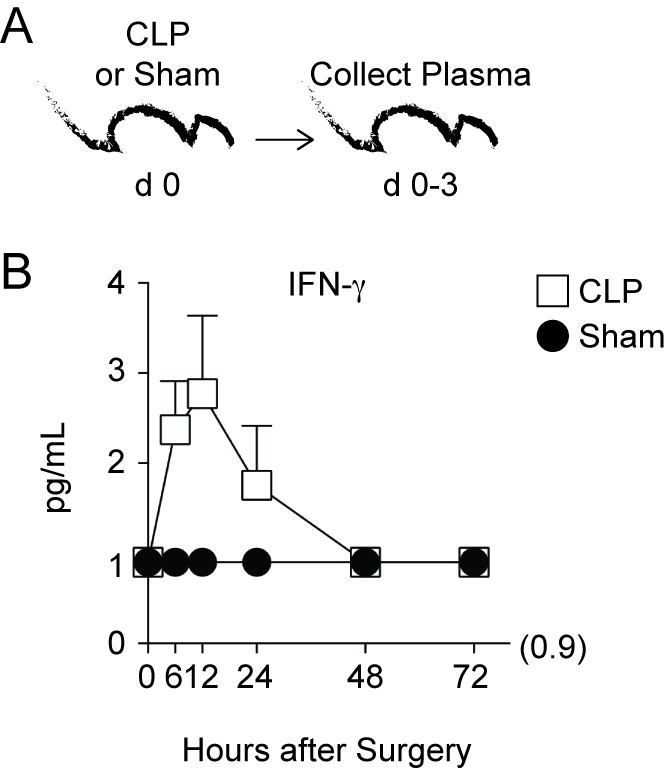

Supplement: S8 Fig — (A) Experimental design. Naive mice received CLP or sham surgery and plasma samples obtained at indicated hours after surgery. (B) Amount of IFN-γ in plasma samples of CLP or sham hosts. LOD of the assay was 0.9 pg/mL. Representative data from two similar experiment with 1–4 mice per group per time point. Error bars represent the standard error of the mean. (TIF) [file ppat.1006569.s008.tif]

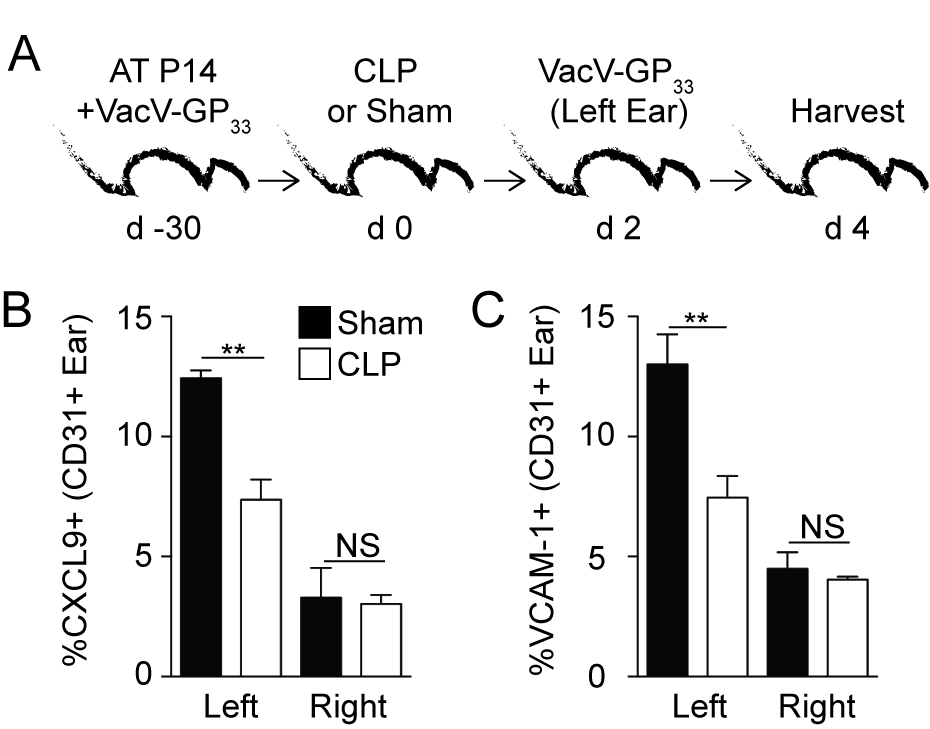

Supplement: S9 Fig — (A) Experimental Design. 8 x 10^3^ naïve P14 CD8 T cells (Thy1.1) were adoptively transferred into C57Bl/6 recipient hosts (Thy1.2) followed by VacV-GP33 infection of the left ear. After 30 days mice underwent CLP or sham surgery and 2 days later mice received homologous VacV-GP33 infection in the same ear. Frequency of (B) CXCL9 and (C) VCAM-1 expression on endothelial cells of the left and right ears 2 days after secondary VacV-infection. Data are representative of two independent experiments with 3–5 mice per group per experiment. NS = not significant; ** = p<0.01. Error bars represent the standard error of the mean. (TIF) [file ppat.1006569.s009.tif]
